# Identification of Key Genes and Pathways for Anaerobic Germination Tolerance in Rice Using Weighted Gene Co-Expression Network Analysis (WGCNA) in Association with Quantitative Trait Locus (QTL) Mapping

**DOI:** 10.1186/s12284-024-00714-y

**Published:** 2024-05-31

**Authors:** Ming Yin, Zhenzhen Zheng, Yue Zhang, Shanwen Wang, Liying Zuo, Yuxin Lei, Yaqiong Zhao, Xiuqin Zhao, Binying Fu, Yingyao Shi, Jianlong Xu, Wensheng Wang

**Affiliations:** 1 State Key Laboratory of Crop Gene Resources and Breeding, Institute of Crop Sciences, Chinese Academy of Agricultural Sciences, Beijing, China; 2 Anhui Agricultural University, Hefei, China; 3 China Agricultural University, Beijing, China; 4 Hainan Yazhou Bay Seed Lab, National Nanfan Research Institute (Sanya), Chinese Academy of Agricultural Sciences, Sanya, China; 5 Southwest United Graduate School, Yunnan University, Kunming, China

**Keywords:** WGCNA, QTL, RNA-seq, Tolerance to anaerobic germination, Haplotype analysis

## Abstract

**Background:**

Rice is one of the most important food crops in the world, and with the development of direct seeding methods for rice, exposure to anaerobic stress has become a major factor limiting its growth.

**Results:**

In this experiment, we tested the tolerance to anaerobic germination of rice varieties NIP and HD84, and they were used as parents to construct a DH (doubled-haploid) population. The transcriptomes of NIP (highly tolerant) and HD86 (intolerant), and their progeny HR (highly tolerant) and NHR (intolerant) were sequenced from normal and anaerobic environments. The differentially-expressed genes (DEGs) were subjected to GO (Gene ontology), KEGG (Kyoto Encyclopedia of Genes and Genomes), and WGCNA analyses. QTL mapping of the DH population identified tolerance to anaerobic germination-related chromosomal segments. The transcriptome results from 24 samples were combined with the anaerobic stress QTL results for 159 DH population lines to construct a metabolic network to identify key pathways and a gene interaction network to study the key genes. Essential genes were initially subjected to rigorous functional validation, followed by a comprehensive analysis aimed at elucidating their potential utility in domestication and breeding practices, particularly focusing on the exploitation of dominant haplotypes.

**Conclusion:**

The results show that pyruvate decarboxylase (PDC) and alcohol dehydrogenase (ADH) are the starting signals of energy metabolism for coleoptile length growth, the auxin transporter EXPA is the determining signal for coleoptile length growth. The pivotal genes *Os05g0498700* and *Os01g0866100* exert a negative regulatory influence on coleoptile length, ultimately enhancing tolerance to anaerobic germination in rice. Analyses of breeding potential underscore the additional value of *Os05g0498700*-hyp2 and *Os01g0866100*-hyp2, highlighting their potential utility in further improving rice through breeding programs. The results of our study will provide a theoretical basis for breeding anaerobic-tolerant rice varieties.

**Supplementary Information:**

The online version contains supplementary material available at 10.1186/s12284-024-00714-y.

## Background

Rice is currently one of the world’s most important food crops, and thus it plays a pivotal role in the lives of billions of people around the world. At present, China is the world’s largest producer and consumer of rice (Xin et al. 2020). In order to save economic costs, efficient and labor-saving methods of rice farming, which include direct sowing of rice seeds, are being developed everywhere (Lv et al. 2021). Compared with traditional cultivation systems, the probability of rice experiencing external stresses in the field after sowing is significantly higher for direct seeding, with anaerobic conditions being the main stress to which direct-seeded rice responds (Mondal et al. 2020). Anaerobic stress affects the uptake of nutrients and water by rice seedlings, causing an imbalance in the energy metabolism of the plant. At this time, high levels of reactive oxygen species (ROS) are produced in plant cells, making the plant unable to grow normally. Solving the issue of anaerobic stress of rice at the germination stage will be highly significant to allow direct-seeded planting of large areas (Yu et al. 2021).

When experiencing anaerobic stress, rice will mitigate the effects of external stress on the plant through a series of measures (Pucciariello 2020). Initially, rice plants up-regulate α-amylase activity and ethanol fermentation efficiency to produce more energy for seedling growth and development (Takahashi et al. 2014; Lee et al. 2014). Secondly, plants begin to regulate levels of phytohormones such as auxin to promote the growth of the coleoptile, which allows the seedling to break through the water surface faster and relieve the anaerobic stress (Nghi et al. 2021). Finally, rice resists oxidative damage from anaerobic stress by regulating the activity of antioxidant substances such as glutathione (Siddiqui et al. 2021). Transcriptome analysis (RNA-seq) is a way to quantify the expression of all expressed genes in different samples, and analyzing the expression of genes in different resistant crops allows for the identification of the relevant resistance mechanisms and key resistance genes (Muhammad et al. 2019). With the development of high-throughput sequencing technology, RNA-seq has been widely used to uncover the molecular mechanisms of stress resistance in rice (Pradhan et al. 2019), cotton (Zhu et al. 2018), wheat (Chaudhary et al. 2021), maize (Zhang et al. 2020), and other crops. Weighted gene co-expression network analysis (WGCNA) is a well-established method in transcriptomics for differential gene analysis in large samples. By constructing a gene co-expression network with a small number of key nodes, it is divided into different modules according to gene expression patterns. The different modules are then associated with the crop phenotypic data to identify the gene modules that are most relevant to the phenotype. Finally, the key genes in the module are determined by analyzing the correlation between genes in the module, which may be the key genes for crop resistance (Langfelder and Horvath 2008).

In addition to transcriptomics, the main method of screening for genes that control resistance traits is QTL mapping, which mainly uses linkage and recombination data for molecular markers and quantitative trait scores to identify functional gene locations (Würschum 2012). The main indicators of QTL in rice for anaerobic resistance include seedling survival, coleoptile length, and bud length. A previous study identified qAG7.1 on chromosome 7, which has a LOD score of 14.5 with 31.7% of the phenotypic variance explained (R^2^), by measuring the survival rate of seedlings in anaerobic condition (Septiningsih et al. 2013). A QTL on chromosome 3 that explained 27% of the phenotypic variation by measuring the coleoptile length (Hsu and Tung 2015).

A target gene identified by a single experimental method has a certain error rate, and the accuracy in identifying key genes by combining RNA-seq with QTL data will be greatly improved (Sevanthi et al. 2021). Derakhshani et al. found 16 candidate genes related to cadmium tolerance in barley using QTL and RNA-seq analyses (Derakhshani et al. 2020). QTL and RNA-seq data were used to identify four candidate genes associated with heat tolerance in tomato (Wen et al. 2019), and also to identify *OsSAP16* as a key salt resistance gene in rice. All of these studies show the validity of combining RNA-seq and QTL analyses for gene mining.

In our study, the *japonica* rice cultivar ‘Nipponbare’ (NIP, more tolerant of anaerobic conditions) and ‘Haidao86’ (HD86, less tolerant of anaerobic conditions) and their hybrid progeny were used in the experiments. The transcriptomic assays were performed on parental and extreme differential progeny plants grown in a normal environment and under anaerobic stress, and the DEGs were subjected to GO, KEGG, and WGCNA analyses. Localization of anaerobic QTLs was performed using a population of 159 DHs derived from a cross between NIP and HD86. Metabolic network suggested that PDC and ADH are the starting signals for coleoptile growth, and that the auxin transporter EXPA is the determining signal for coleoptile length growth. We have identified that *Os05g0498700* and *Os01g0866100* possess the capability to augment tolerance to anaerobic germination in rice. Through assessments grounded in domestication and evolutionary insights, it is evident that the predominant haplotypes of these genes hold considerable breeding potential. These results provide novel resources for improving the mechanism of anaerobic stress tolerance and the development of rice cultivars that are tolerant of anerobic conditions.

## Results

### Determination of Coleoptile Length, Glycoconjugate Contents, and Enzyme Activities in the Parental Cultivars

**Fig. 1:**
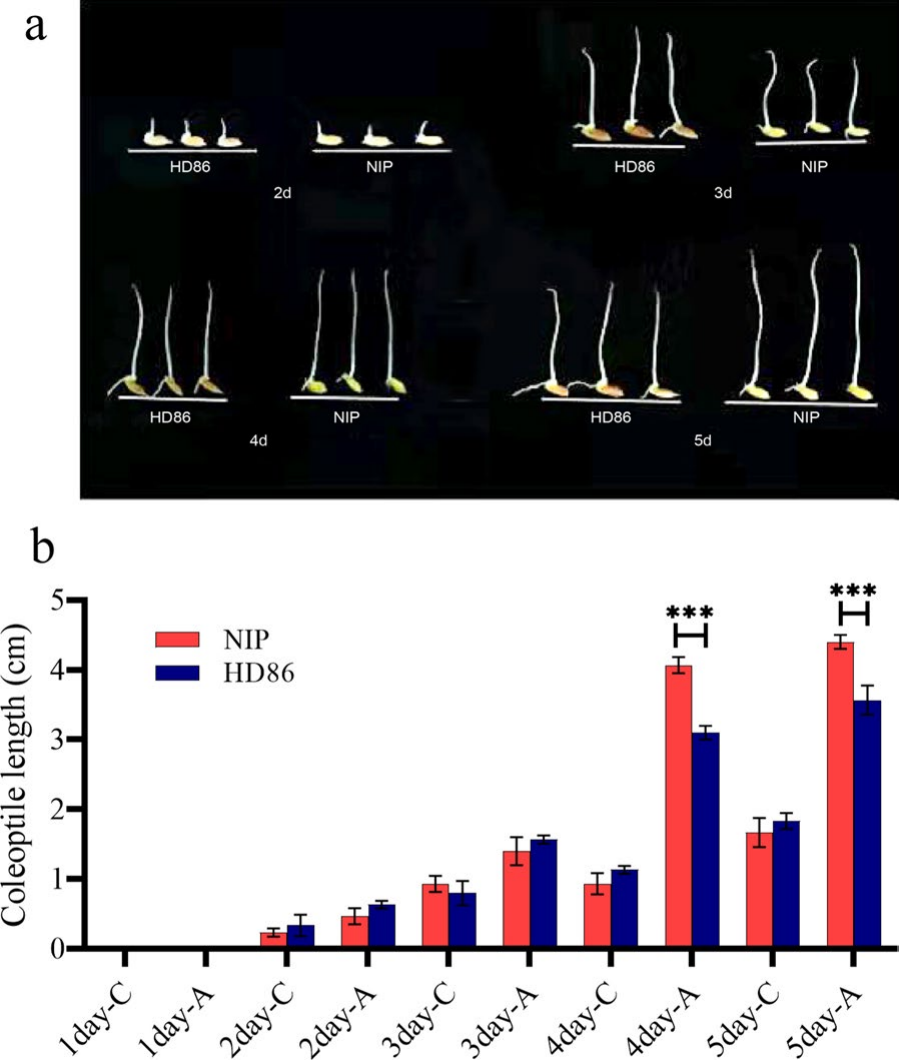
Phenotypic variation of coleoptile in HD86 and NIP under anaerobic (hypoxic) conditions. **a** Coleoptile growth in HD86 and NIP under anaerobic (hypoxic) conditions. **b** Mean coleoptile lengths from days 1 to 5 in HD86 and NIP seeds germinated under anaerobic conditions and normal (control) conditions. A indicates anaerobic stress, **C** indicates control conditions

To determine the tolerance to anaerobic germination of HD86 and NIP, the lengths of the coleoptiles were compared at different times under anaerobic stress versus normal growth conditions. The oxygen concentrations in the anaerobic and normal environments were 2.17 and 10.8 mg/L, respectively. When comparing the coleoptile lengths of the two varieties, no significant differences were observed under normal conditions. The greatest difference in coleoptile length between the two lines was observed on day 4 of anaerobic stress (Fig. 1a), and the NIP coleoptile was significantly longer than that of HD86, with a significant difference of 0.97 cm (Fig. 1b).

**Fig. 2:**
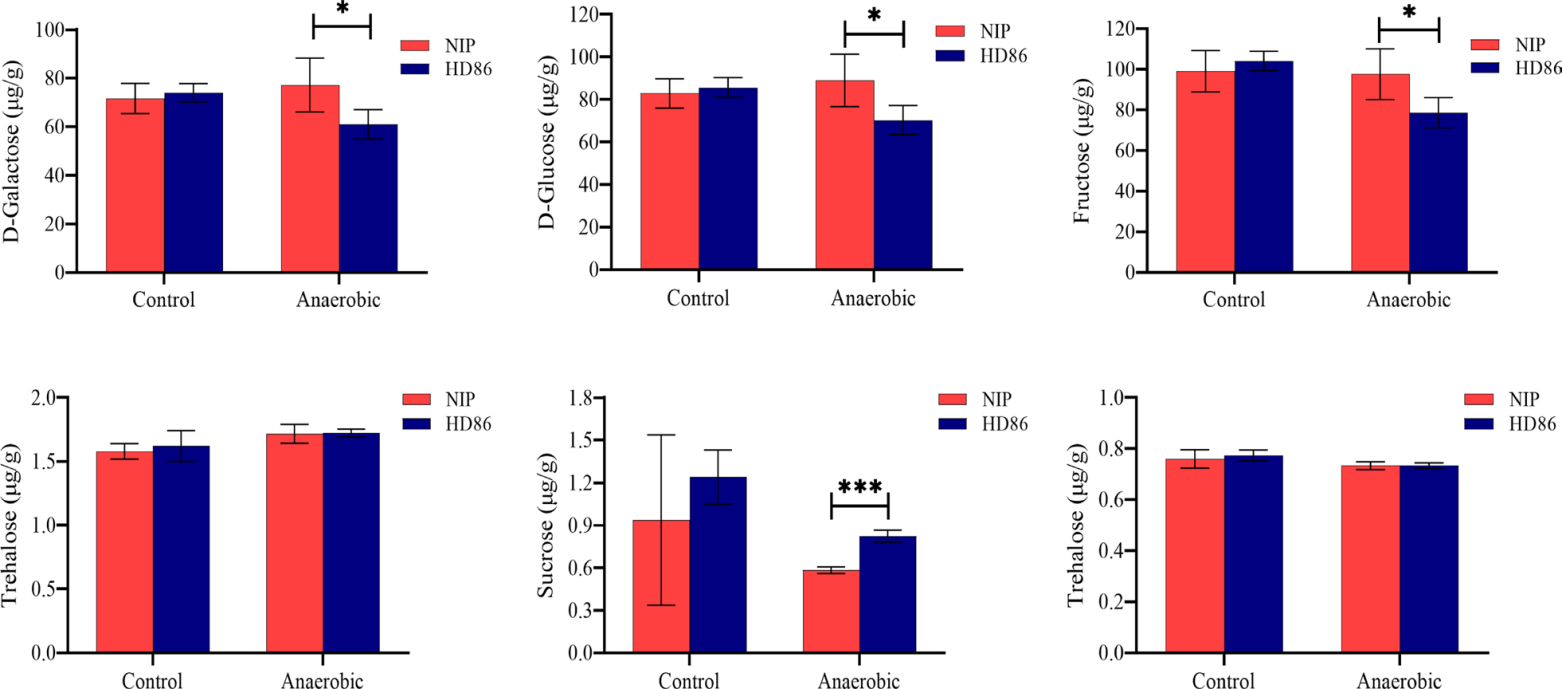
Contents of six disaccharides in the coleoptiles of HD86 and NIP

In this study we measured the contents of six major glycoconjugates (Table S1) and the activities of α-amylase (Fig. S1a) and pyruvate decarboxylase (Fig. S1b) in HD86 and NIP in the two environments (control and anaerobic). For the six disaccharides (Fig. 2), the maltose content increased more in NIP; d-glucose and D-galactose decreased in HD86 and increased in NIP; fructose, trehalose, and sucrose decreased in both cultivars, but the decrease was less in NIP. Comparing the normal and anaerobic environments, α-amylase activity increased by 3.79 nmol/min/g in HD86 and 5.29 nmol/min/g in NIP, and pyruvate decarboxylase activity decreased by 41.18 nmol/min/g in HD86 and 18.01 nmol/min/g in NIP (Fig. S1).

These results showed that in rice exposed to anaerobic stress, the coleoptile elongates rapidly and its energy metabolism would therefore be significantly affected. The sugars and related enzymes in the NIP coleoptile were more positively regulated in NIP compared with HD86, indicating that NIP has stronger energy metabolism under anaerobic stress. Among the disaccharide sugars, d-glucose, fructose, and d-galactose may be the key glycoconjugates that determine differential stress tolerance in the two rice varieties.

### Transcriptome Sequencing Information and Bioinformatics Analysis of the DH Population Parental Cultivars

Sequencing mRNA from the parental lines gave 31,247,174–43,427,818 clean reads, and the percentage of high-quality bases (Q30) were > 92.14%, from all samples, with a mapping range of 91.49%-97.88% and a unique mapping range of 96.04%-97.55% (Table S2). Cluster analysis of the biological replicates was performed on each sample based on transcriptome results, and the results showed a clear separation of the different cultivars and a clear clustering of the same cultivars, indicating that the data is highly reliable (Fig. S2a).

**Fig. 3:**
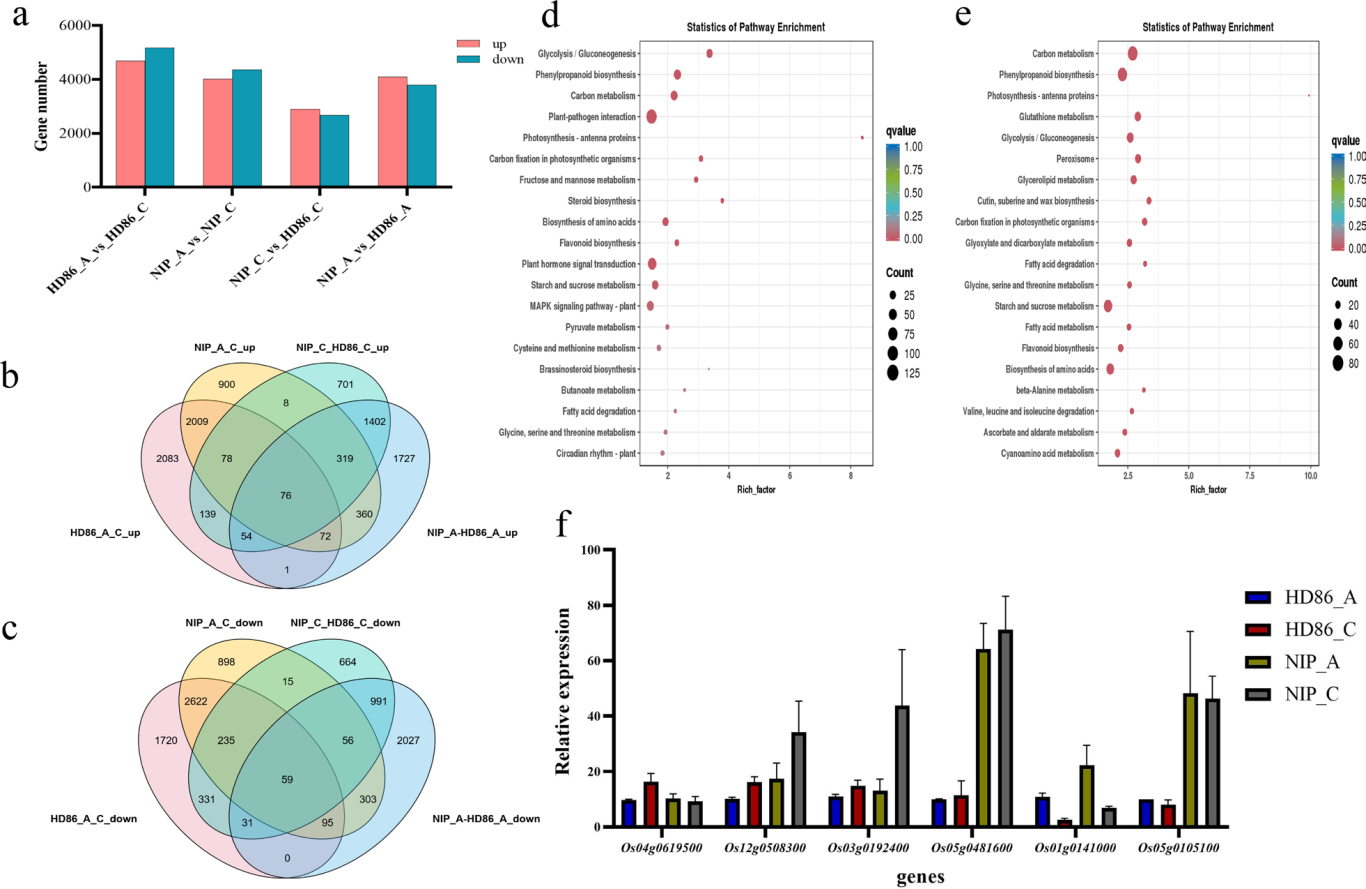
RNA-seq analysis of HD86 and NIP and qRT-PCR validation. **a** The number of differentially expressed genes (DEGs) in the four parental line comparisons. **b** Veen diagram of parental up-regulation genes. **c** Veen diagram of parental down-regulation genes. **d** KEGG enrichment results of parental up-regulated genes. **e** KEGG enrichment results of parental down-regulated genes. **f** qRT-PCR analysis of gene expression for six DEGs from the parental transcriptome

The DEGs between the two parental lines were identified based on their gene expression levels (Fig. 3a). GO term analysis comparing HD86_A (anaerobic stress) and HD86_C (normal conditions), shown in Fig. S2b, revealed functions such as ‘transition metal ion transmembrane transporter activity’, ‘cellular response to hormone stimulus’, and ‘cellular response to auxin stimulus’, amongst others. Likewise, the comparison between NIP_A and NIP_C (Fig. S2c) highlighted GO terms including ‘calmodulin binding’, ‘cellular response to hormone stimulus’, and ‘cellular response to auxin stimulus’. Additionally, the KEGG enrichment analysis for HD86_A vs. HD86_C (Fig. S2d) emphasized pathways like ‘Glycolysis’, ‘RA biosynthesis pathway’, and ‘Signaling by Retinoic Acid’. For NIP_A vs. NIP_C (Fig. S2e), enriched pathways comprised ‘Alpha-oxidation of phytanate’, ‘Vitamins’, and ‘Peroxisomal lipid metabolism’.

Furthermore, we examined genes that exhibited consistent up-regulation (Fig. 3b) or down-regulation (Fig. 3c) across various groups and conducted KEGG enrichment analysis on them. Notably, the consistently upregulated genes (illustrated in Fig. 3d) were primarily enriched in ‘Photosynthesis-antenna proteins’, ‘Steroid biosynthesis’, and ‘Glycolysis/Gluconeogenesis’. In contrast, the consistently downregulated genes (depicted in Fig. 3e) were predominantly enriched in ‘Photosynthesis-antenna proteins’, ‘Cutin, suberine, and wax biosynthesis’, and ‘Carbon fixation in photosynthetic organisms’. We selected six DEGs from the transcriptome and quantified their relative expression using qRT-PCR. The results corroborated the transcriptome data, confirming its reliability (Fig. 3f).

### Transcriptome Sequencing Information and Bioinformatics Analysis of DH Population Progeny

Sequencing of the progeny plants produced 45,237,706–49,025,002 clean reads, of which > 95.2% were high-quality bases (Q scores ≥ Q30) with a mapping range of 95.17%-96.40% and a unique mapping range of 96.71%-97.55% (Table S3). Biological replicate cluster analysis showed this data to be highly reliable (Fig. S3a).

**Fig. 4:**
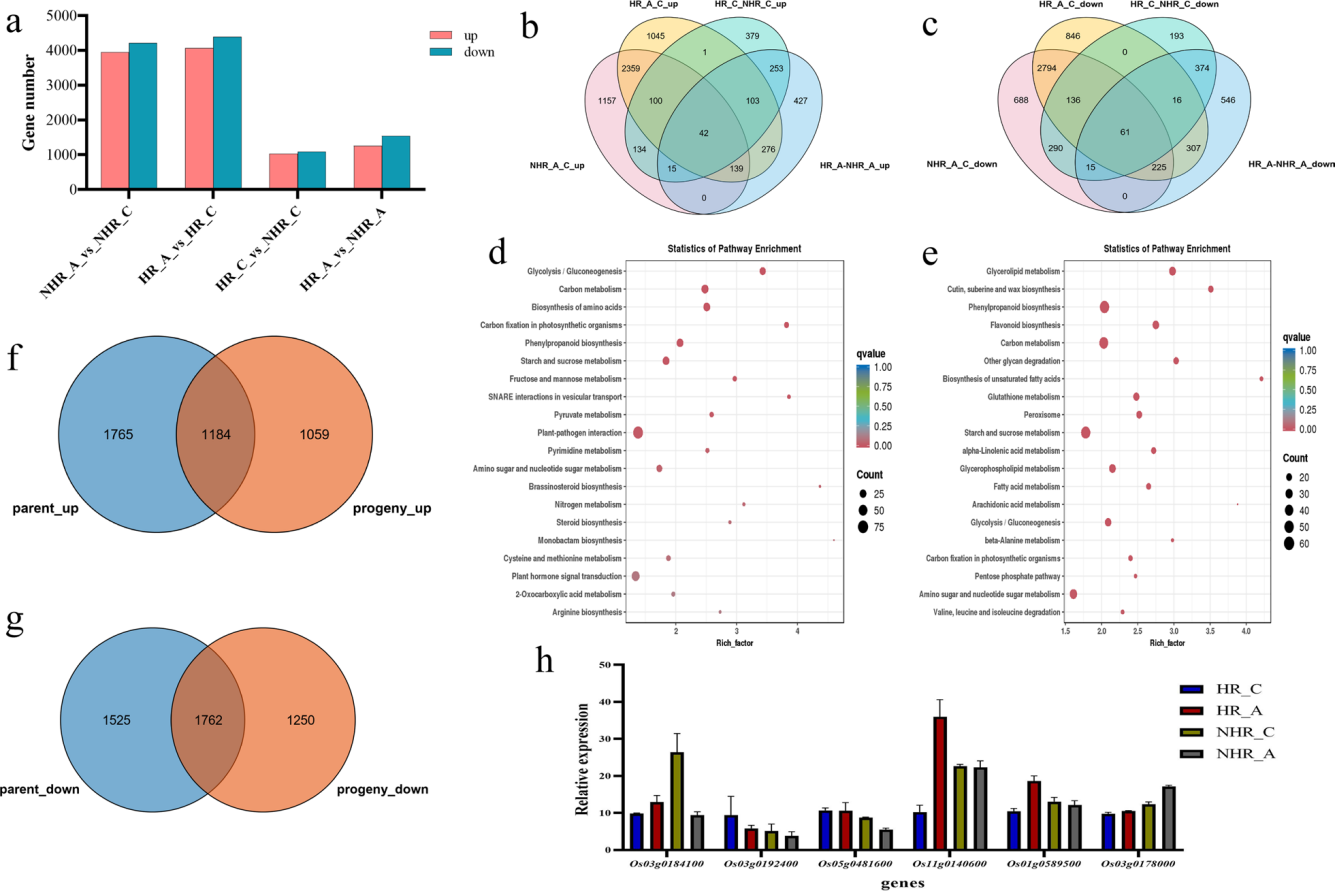
RNA-seq analysis of HR and NHR and qRT-PCR validation. **a** The number of differentially expressed genes (DEGs) in the four progeny comparisons. **b** Veen diagram of progeny up-regulation genes. **c** Veen diagram of progeny down-regulation genes. **d** KEGG enrichment results of progeny up-regulated genes. **e** KEGG enrichment results of progeny down-regulated genes. **f** Veen diagram of up-regulated genes in parents and offspring. **g** Veen diagram of down-regulated genes in parents and offspring. **h** qRT-PCR analysis of gene expression for six DEGs from the second transcriptome

DEGs in progeny comparisons were identified through gene expression analysis (Fig. 4a). GO enrichment analysis showed significant DEG differences in HR_A vs. HR_C (Fig. S3b) and NHR_A vs. NHR_C (Fig. S3c) comparisons. KEGG enrichment also revealed distinct patterns for these comparisons (Fig. S3d, e). We compared consistently up- or down-regulated genes (Fig. 4b, c) across groups and conducted KEGG enrichment on them. Up-regulated genes (Fig. 4d) were enriched in processes like ‘Carbon fixation in photosynthetic organisms’, ‘Glycolysis, Gluconeogenesis’, ‘Fructose and mannose metabolism’, while down-regulated genes (Fig. 4e) were enriched in ‘Biosynthesis of unsaturated fatty acids’, ‘Cutin, suberine and wax biosynthesis’, ‘Glycerolipid metabolism’. Many differential genes were linked to energy metabolism and secondary metabolite synthesis in both parents and progeny.

Based on anaerobic stress phenotypes, we screened for consistently trending genes, finding 1184 up-regulated (Fig. 4f) and 1762 down-regulated genes (Fig. 4g). These can serve as a reference for studying rice hypoxia stress tolerance (Table S4). qRT-PCR assays on six DEGs confirmed the transcriptome data's reliability (Fig. 4h).

### WGCNA Analysis of the Transcriptomes

**Fig. 5:**
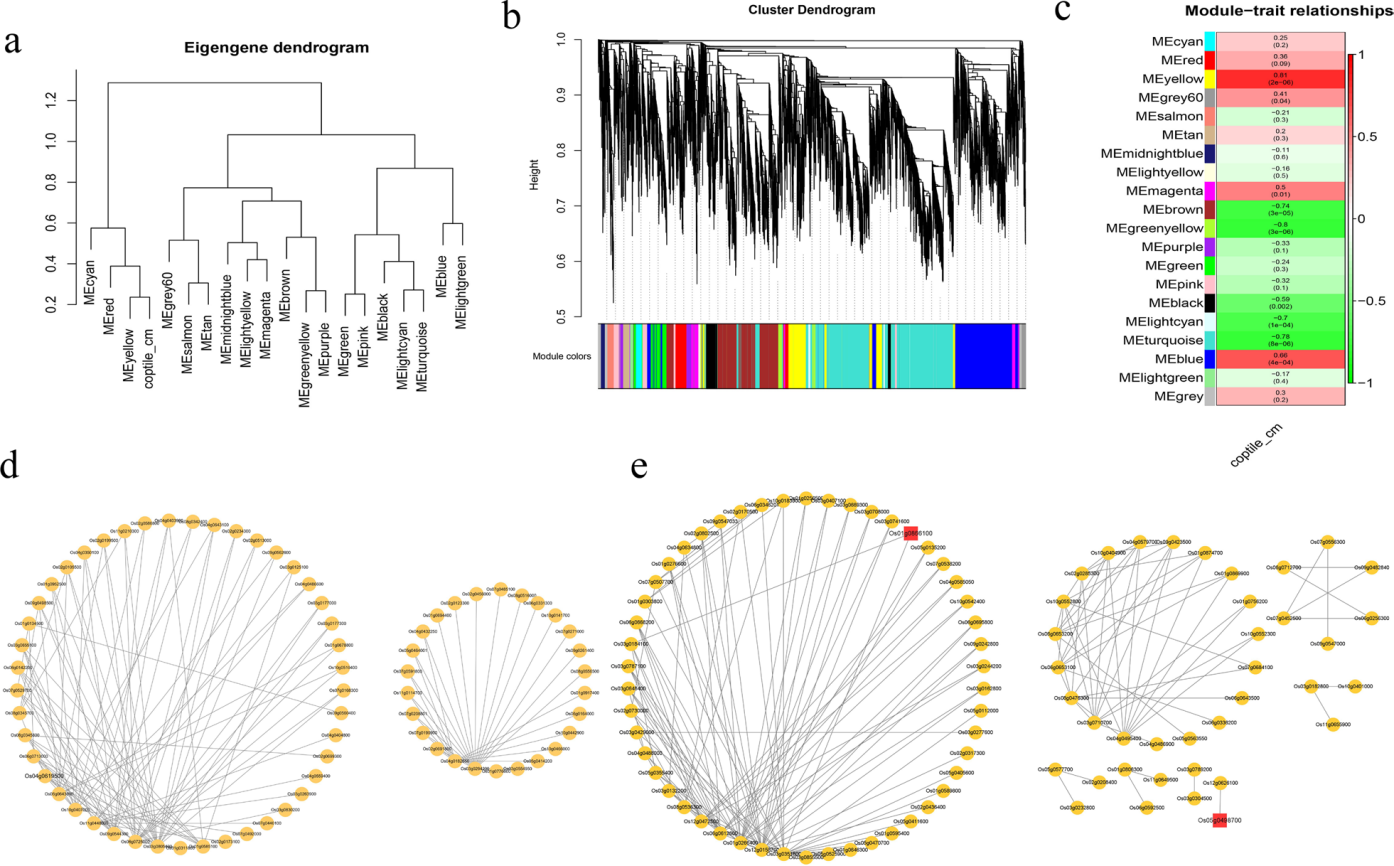
WGCNA of genes identified in 24 transcriptome samples. **a** Clustering of the 24 transcriptome samples from the parental cultivars (HD86 and NIP) and the DH progeny (HR and NHR) under normal and anaerobic conditions. **b** Construction of the different modules. **c** Correlation between each module and rice coleoptile length. **d** Gene association network of the MEblue module. **e** Gene association network of the MEyellow module

The DEGs from in the 24 transcriptome samples were analyzed by WGCNA to identify the genes most associated with tolerance to anaerobic germination in rice. We first performed screening for outliers (Fig. 5a), and none of the 24 samples was found to have outliers and all could be used in the subsequent analysis. A threshold value of 14 was determined for constructing a scale-free network (Fig. S4a), which will divide the different genes into different modules based on their expression (Fig. 5b). Since the length of the rice coleoptile is a marker of tolerance to anaerobic germination in rice, each module was correlated with the length of the rice coleoptile (Fig. 5c), and a correlation heatmap was constructed for all modules (Fig. S4b). The results showed that the MEyellow and MEblue modules were significantly and positively correlated with the coleoptile length in rice, with values of 0.81 and 0.66, respectively. The two modules contained 1,127 (MEblue) and 532 (MEyellow) genes. The genes in the MEblue (Fig. 5d) and MEyellow (Fig. 5e) modules were networked according to their interrelationships using cytoscape software, and the genes with high connectivity were selected as candidates for key genes based on the ranking of the connectivity between genes.

### Population Phenotype Data Statistics, Construction of a Genetic Linkage Map, and QTL Localization

**Fig. 6:**
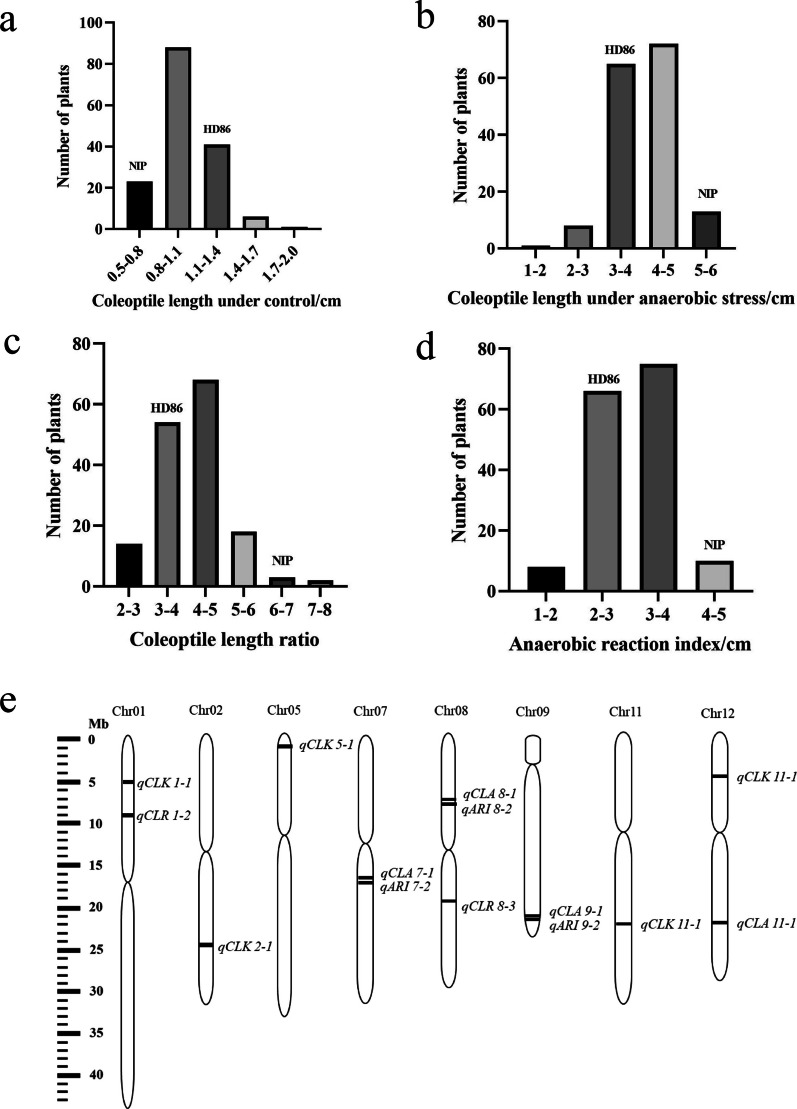
QTL-seq analysis of the parental cultivars (HD86 and NIP) and the DH population (HR and NHR). Distribution of coleoptile length in the parental cultivars (HD86 and NIP) and the DH population (HR and NHR) under **a** control conditions and **b** under anaerobic stress. **c** Coleoptile length ratio in HD86 and NIP and the DH population. **d** Anaerobic reaction index in HD86 and NIP and the DH population. **e** Genetic linkage map of the 12 rice chromosomes showing the positions of QTLs associated with anaerobic phenotypes in the DH population

Phenotypic data were recorded between the parents and the 159 DH lines; under normal conditions, coleoptile length in the DH population ranged from 0.5 to 1.9 cm, and the lengths for HD86 and NIP were 1.2 and 0.8 cm, respectively (Fig. 6a). Under anaerobic stress, coleoptile length ranged between 1.8 and 5.6 cm for the DH population, and the values were 4.0 cm for HD86 and 5.2 cm for NIP (Fig. 6b). Under normal conditions, the radicle length ratio ranged from 2.6 to 7.4 in the DH population, and the ratios were 3.3 for HD86 and 6.5 for NIP (Fig. 6c). The range of the anaerobic response index was 1.1–4.7 cm in the DH population, 2.8 cm for HD86, and 4.3 cm for NIP (Fig. 6d).

From 40 K SNPs, we screened 20,158 high quality SNP markers that showed polymorphism between the parental cultivars, and 2,842 SNP markers were obtained by removing redundant markers. We constructed chromosomal linkage maps with a total length of 1,491.8 cM (Fig. 6e), and performed the QTL localization. A total of 14 QTLs associated with hypoxia were located on chromosomes 1, 2, 5, 7, 8, 9, 11, and 12 by correlation statistics of phenotypic data (Table S5). The qCLR 1–2 interval (53,515 bp) includes the cloned semi-dwarf gene *sd1*, which can suppress plant height in rice and is one of the key genes that featured in the Green Revolution of rice.

### Analysis of Key Metabolic Pathways for Tolerance to Anaerobic Germination in Rice

**Fig. 7:**
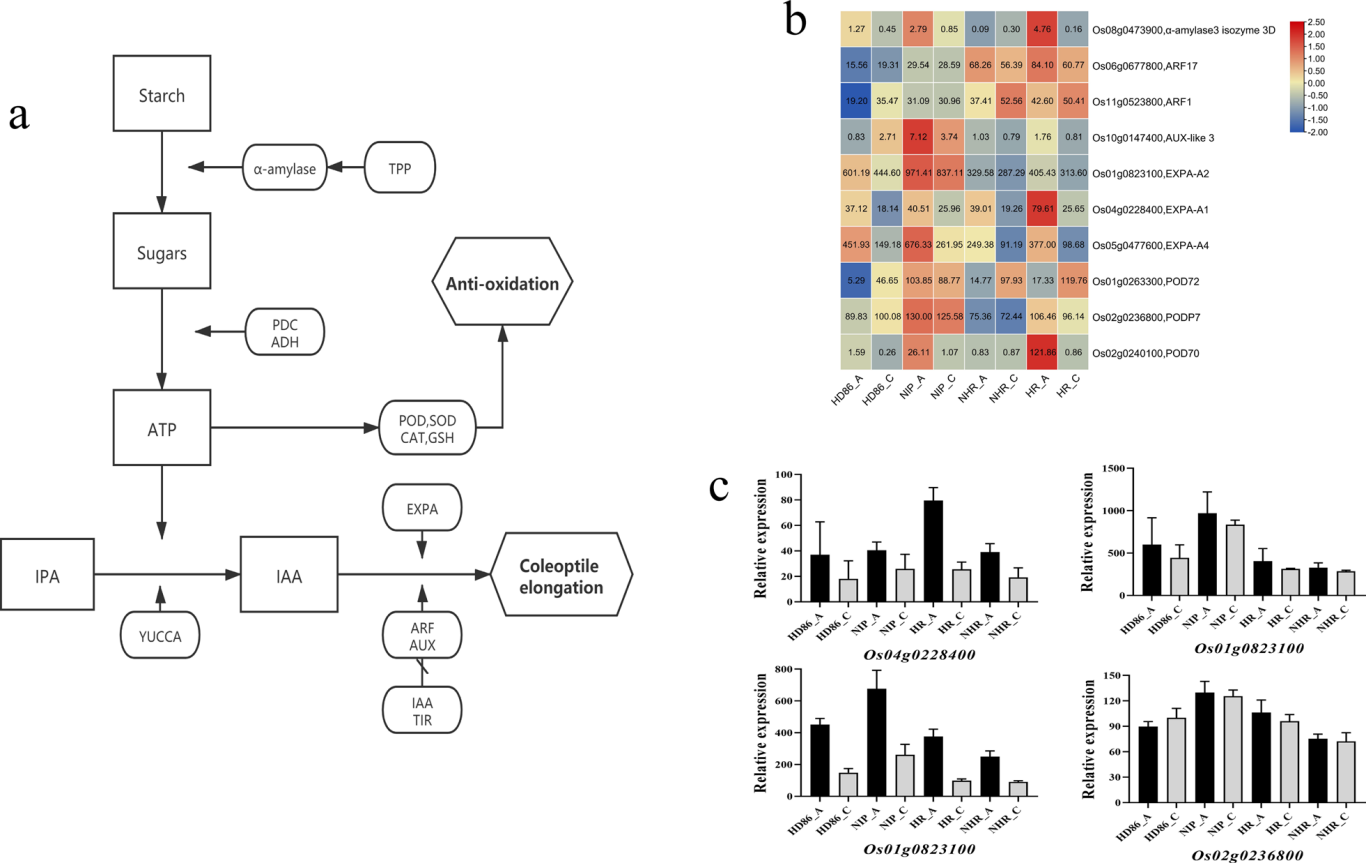
Proposed mechanism of candidate genes function in enhancing anaerobic germination. **a** The tolerance to anaerobic germination regulatory network in rice. **b** Heatmap showing the expression of 10 key genes from the network in the parental and DH progeny comparisons from the control and anaerobic treatments. **c** Histogram of 4 DEGs from 10 key genes

The 456 genes localized to the 19 QTL intervals were compared for gene expression; the qARI 9-2 interval (~ 1.45 Mbp) was not analyzed because it was too large. The genes with large differences in expression belonged mainly to pathways involving energy metabolism, phytohormones, and antioxidant substances. Based on the results of QTL analysis and GO and KEGG enrichment of the transcriptome, we constructed a related regulatory network for rice seeds (Fig. 7a). Among the genes, Half of the *TPP* gene (Fig. S5a) expression was up-regulated and half down-regulated*. α-AMY* (Fig. S5b) encodes a key enzyme for catabolism of starch, and its expression showed a decreasing trend after anaerobic stress; however, the expression of *Os08g0473900* (α-amylase3 isozyme 3D) increased substantially in NIP, and was higher than in HD86. *PDC* (Fig. S5c) and *ADH* (Fig. S5d) are key genes for ATP (Adenosine triphosphate) production after *α-AMY*, and the magnitude of the changes in expression of these genes was more pronounced in HD86 and NHR after stress.

*YUCCA* (Fig. S6a), *ARF* (Fig. S6b), *AUX* (Fig. S6c), *IAA* (Fig. S6d), *TIR* (Fig. S6e), and *EXPA* (Fig. S6f)-related genes are involved in the growth of the radicle, of which *YUCCA*-like genes are important for growth hormone production, but they do not show specific expression patterns in response to anaerobic stress. *ARF* and *AUX* are genes that act downstream of growth hormones. Most *ARF* gene expression showed a downward trend after anaerobic stress, but *Os06g0677800* (*ARF17*) and *Os11g0523800* (*ARF1*) were up-regulated after stress in NIP and HR, and the magnitude was greater than in HD86 and NHR. *IAA* and *TIR* are genes that are negatively regulated by *AUX*, where the post-stress up-regulation of *IAA* genes is higher in NIP and HR, corresponding to the smaller increase in *AUX* expression in NIP, which confirms the reliability of the regulatory network and transcriptome data. *EXPA* genes encode growth hormone transport proteins, and the expression of *EXPA* genes increases more in NIP than in HR, and *Os01g0823100* (*EXPA-A2*), *Os04g0228400* (*EXPA-A1*), and *Os05g0477600* (*EXPA-A4*) may be key genes involved in the elongation of the coleoptile.

Genes for superoxide dismutase (SOD, Fig. S7a), peroxidase (POD, Fig. S7b), catalase (CAT, Fig. S7c), and glutathione (GSH, Fig. S7d) all encode proteins that function in detoxifying reactive oxygen species (ROS). All of the *SOD*-like genes showed decreased expression after stress, but the relative decrease was smaller in the NIP varieties. In contrast, expression of *POD* genes was substantially increased in both NIP and HR varieties, and the up-regulation was generally higher in HR than in NHR. Among the *POD* genes, the expression of *Os01g0263300* (*POD72*), *Os02g0236800* (*PODP7*), and *Os02g0240100* (*POD70*) was up-regulated under anaerobic stress in NIP and HR. The expression of both *CAT* and *GSH* genes decreased, and the changes were similar in all varieties with no significant differences. The metabolic network and the related gene expression enabled us to identify some key genes in the network (Fig. 7b, c).

### Identification of Key Genes for Tolerance to Anaerobic Conditions (Hypoxia) in Rice

**Fig. 8:**
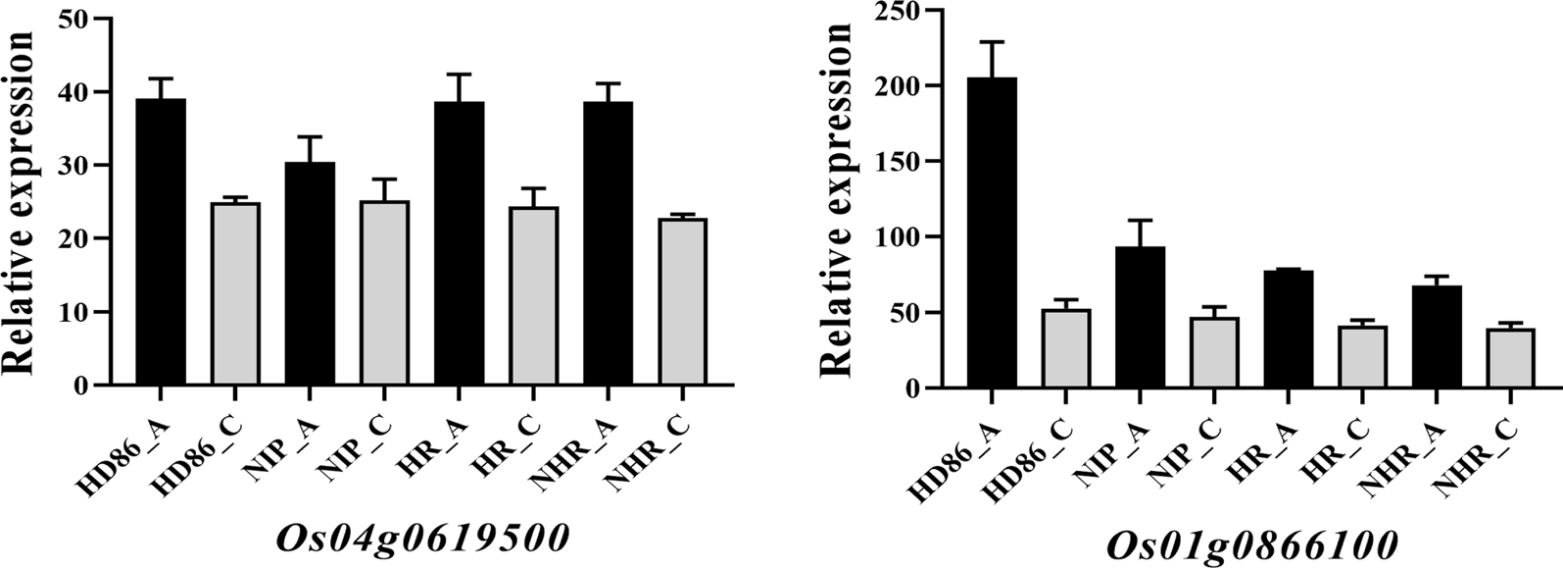
Histogram of three key genes identified from the WGCNA and QTL mapping

**Fig. 9:**
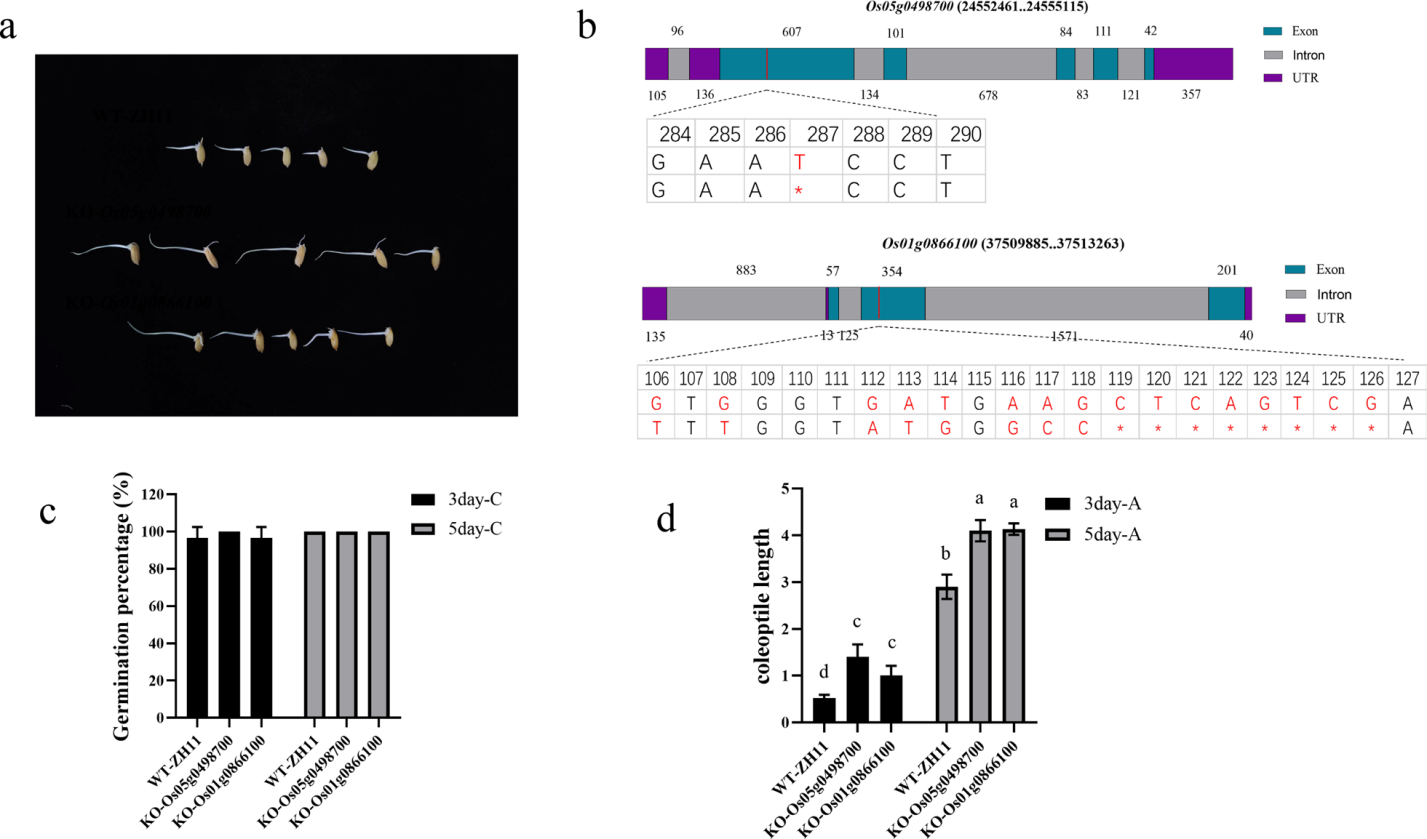
Candidate gene mutant creation and phenotyping. **a** The coleoptile of key genes mutants at 3 days under anaerobic stress. **b** Gene structure and mutant information for Candidate gene. **c** Germination rate of candidate gene mutants under Normal environmental. **d** The coleoptile length under anaerobic stress of Candidate gene mutant

**Fig. 10:**
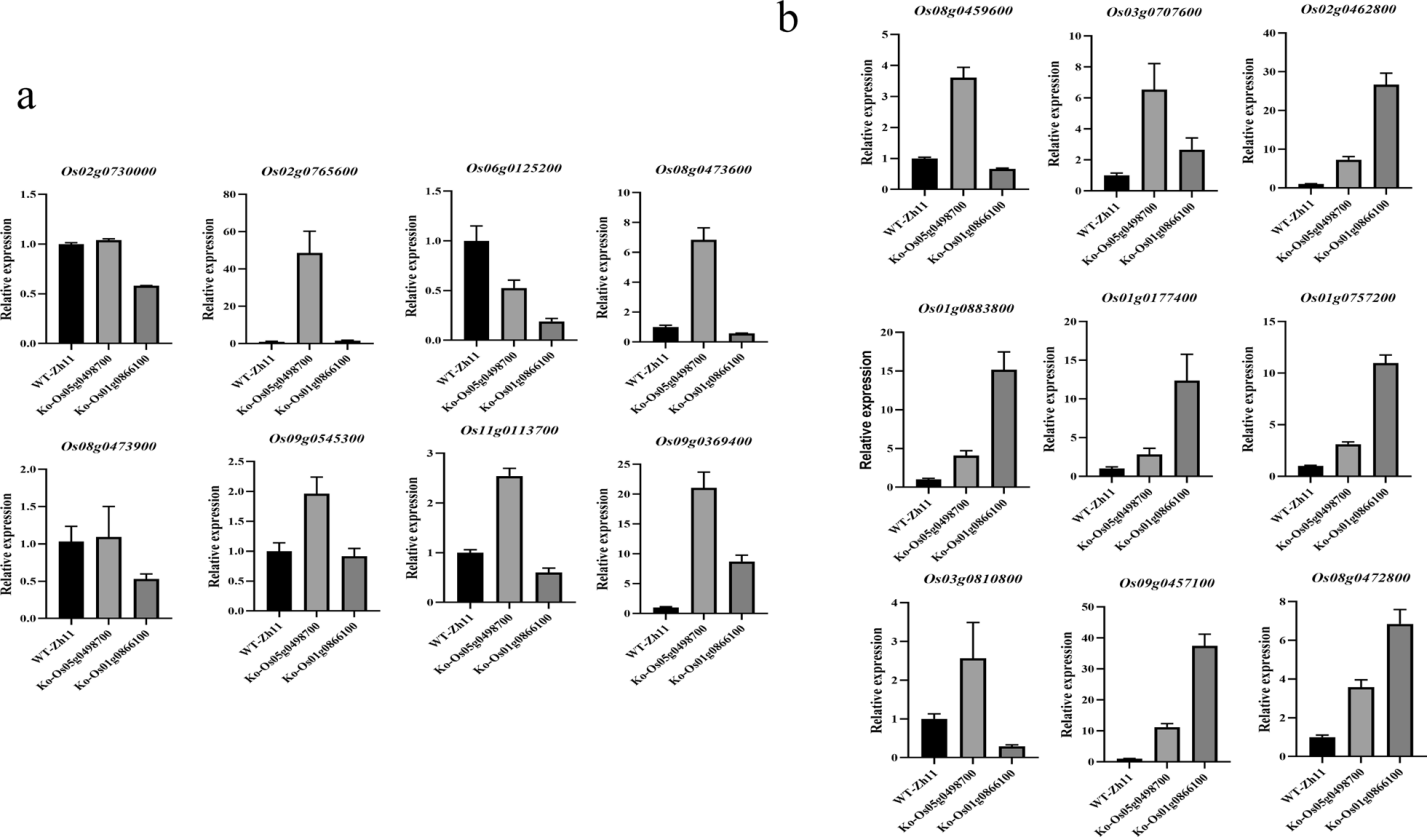
Gene expression in coleoptile under anaerobic stress in candidate gene mutants. **a** Expression of cloned coleoptile-related genes. **b** Expression of cloned plant hormone-related genes

The results of transcriptome WGCNA analysis were compared with the QTL results to identify key genes for tolerance to anaerobic germination in rice. The top 50 genes with the highest connectivity in the two modules in the WGCNA network were selected and compared with the genes localized by QTL analysis; *Os05g0498700* and *Os01g0866100* were found to be common to both sets of results. The expression of all three genes was up-regulated after stress (Fig. 8). Knockout mutants were generated for three candidate genes (Fig. 9a–b). In comparison to the wild type ZH11, there was no notable difference between the *Os05g0498700* and *Os01g0866100* mutants under normal conditions (Fig. 9c). Subsequently, the coleoptile length of the experimental materials was assessed under anaerobic stress conditions. The *Os05g0498700* and *Os01g0866100* mutants displayed significantly longer coleoptiles than the ZH11 (Fig. 9d). To elucidate the impact of *Os05g0498700* and *Os01g0866100* on rice coleoptile growth, we conducted an assessment of the expression levels of anaerobic stress-related genes and plant hormone-related genes in the coleoptiles of the respective mutants (Fig. 10). The results demonstrated a substantial upregulation in gene expression levels of *Os02g0765600* (α-amylase), *Os08g0473600* (α-amylase), and *Os09g0369400* (trehalose-6-phosphophosphatase) in the *Os05g0498700* mutant, exhibiting a remarkable five-fold increase compared to their wild-type counterparts under anaerobic stress conditions. In contrast, in *Os01g0866100* mutants, the expression levels of *Os06g0125200* (a zinc finger protein, known for its negative regulation of coleoptile growth) and *Os09g0369400* (trehalose-6-phosphate phosphatase) also experienced a more than five-fold change, while the expression levels of α-amylase-related genes remained relatively stable (Fig. 10a). Furthermore, we investigated the expression levels of plant hormones, specifically jasmonic acid (JA), gibberellin (GA), and abscisic acid (ABA), which are known to potentially impact coleoptile length growth. Within the JA-related genes, the expression of JA synthesis genes (Fig.   10b–1, 2) exhibited a 3- to sixfold upregulation in *Os05g0498700* mutants, while the change was not statistically significant in *Os01g0866100* mutants. Concurrently, the JA breakdown gene saw a substantial sixfold upregulation in the *Os05g0498700* mutant and an impressive 25-fold upregulation in the *Os01g0866100* mutant ( Fig.  10b-3). Regarding GA-related genes, GA synthesis genes showed a 3- to fivefold upregulation in *Os05g0498700* mutants and an even more pronounced 10- to 15-fold increase in *Os01g0866100* mutants (Fig.   10b-4, 5, 6). In terms of ABA-related genes, the ABA synthesis gene exhibited a 2- to threefold upregulation in the *Os05g0498700* mutant and a substantial fivefold increase in the *Os01g0866100* mutant (Fig. 10b-7). The ABA degradation gene displayed a noticeable 4- to tenfold upregulation in the *Os05g0498700* mutant and a remarkable 6- to 40-fold increase in the *Os01g0866100* mutant (Fig.  10b-8, 9).

### Domestication and Breeding Utilization of Key Gene Dominance Haplotypes

**Fig. 11:**
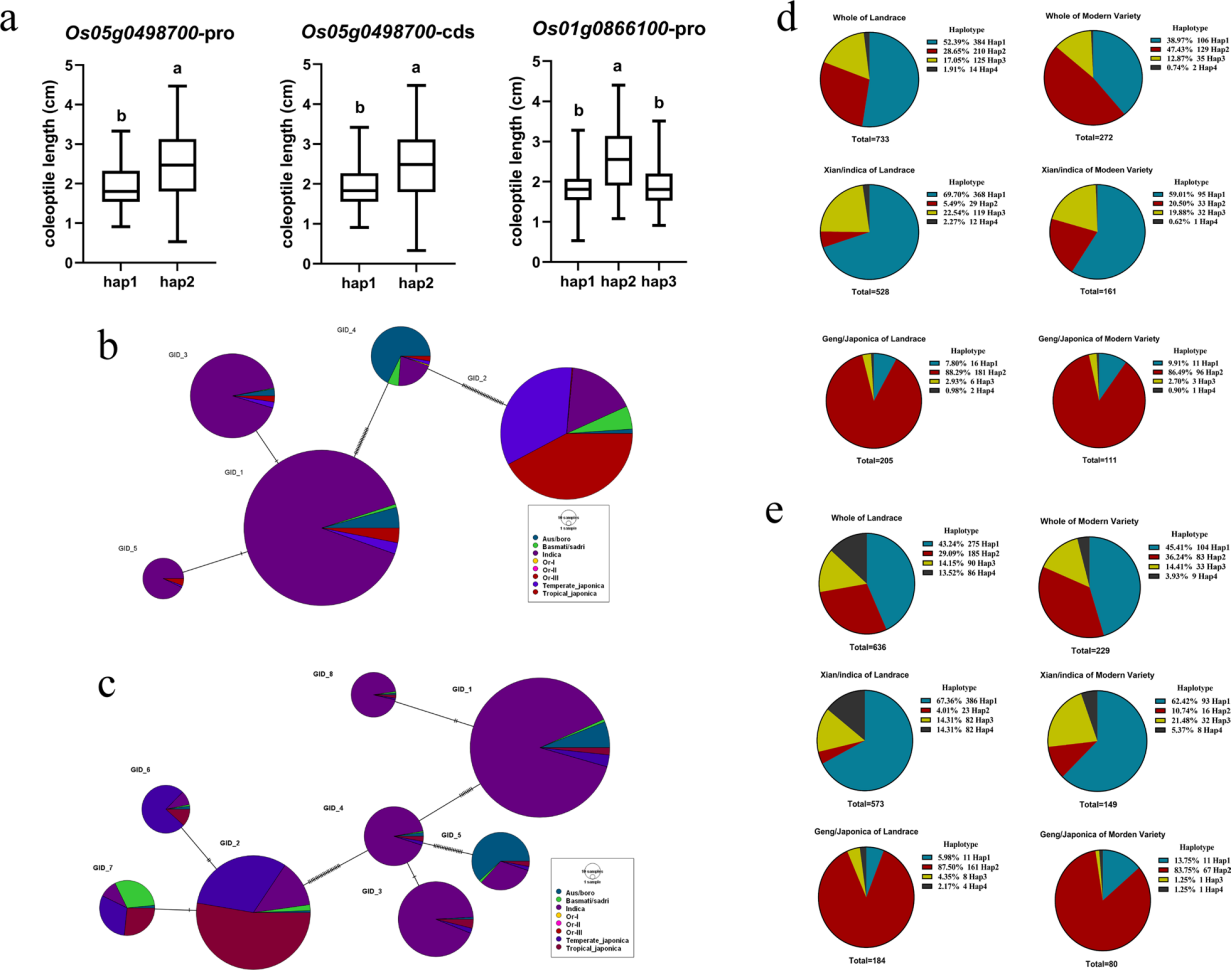
Domestication and breeding exploitation of dominant haplotypes of key genes. **a** Identification of candidate gene dominant haplotypes. **b** Analysis of dominant haplotype domestication of *Os05g0498700*. **c** Analysis of dominant haplotype domestication of *Os01g0866100*. **d** Analysis of dominant haplotype breeding utilization of *Os05g0498700*. **e** Analysis of dominant haplotype breeding utilization of *Os01g0866100*

Haplotypes for *Os05g0498700* and *Os01g0866100* were examined in correlation with coleoptile lengths, utilizing a dataset of 498 diverse rice accessions (Fig. 11a). To shed light on the evolutionary origin of dominant haplotypes in *Os05g0498700* and *Os01g0866100*, we leveraged 3 k sequencing data and data from wild rice sources (Or-1 (18), Or-2 (53), Or-3 (47)) accessible on the MBKbase website for SNP identification. Haplotype network analysis indicated that the dominant haplotype of *Os05g0498700* may have derived from Or-3 (Fig. 11b). However, the dominant haplotype of *Os01g0866100* could not be identified in wild rice (Fig. 11c). To assess whether *Os05g0498700* and *Os01g0866100* underwent selection during domestication (Table S6), we calculated the average nucleotide diversity (π/pi) and Tajima’s D for a 40 kb genomic region centered on *Os05g0498700*. Nucleotide polymorphism within the 20 K upstream region of the gene (0.00049) was significantly lower in japonica rice compared to other subgroups. Additionally, Tajima’s D (2.67182) within the gene body of japonica rice was significantly higher than that of other subgroups. Concerning *Os01g0866100*, Tajima’s D (3.17175) within the gene body of japonica rice also exhibited a significant elevation compared to other subgroups. These findings suggest that both genes underwent varying degrees of selection pressure, and their superior haplotypes have been widely integrated into japonica rice germplasm, possibly representing favorable alleles for enhancing rice’s tolerance to hypoxia.

We investigated the allele frequencies of *Os05g0498700* (Fig. 11d) in a dataset consisting of 1005 cultivated rice germplasms, and similarly, the allele frequencies of *Os01g0866100* in a dataset of 865 cultivated rice germplasms. Our aim was to understand how these two genes are employed in the breeding process. In the course of rice breeding and improvement, we observed changes in the frequency of haplotype 2 (hyp2, dominant haplotype) of the *Os05g0498700*, which increased from 28.65% (in landrace varieties) to 47.43% (in modern varieties). Conversely, the primary haplotype 1 (hyp1) decreased from 53.39% (in landrace varieties) to 38.97% (in modern varieties) across all cultivated rice. In the indica subgroup, the proportion of hyp2 rose from 5.49% to 20.50%, while hyp1 decreased from 69.70% to 59.01%. In the japonica rice subpopulation, the proportion of hyp2 remained relatively stable, possibly due to this haplotype already exceeding 85%, making further increases challenging. For *Os01g0866100* (Fig. 11e), the frequency of hyp2 increased from 29.09% to 36.24% across all cultivated rice, with the most significant decline observed in hyp4 haplotype, decreasing from 13.52% to 3.93%. In the indica subgroup, hyp2 increased from 4.01% to 10.74%, while hyp4 decreased from 14.31% to 5.37%. In the japonica rice subpopulation, the proportion of hyp2 did not exhibit significant changes.

## Discussion

Anaerobic stress is the most common abiotic stress that occurs in direct-seeded rice. With the development of mechanized seeding, studying the metabolic pathways and key genes related to anaerobic stress tolerance in rice can provide a theoretical basis for breeding varieties of rice that tolerate hypoxia. The network of energy metabolism, phytohormone metabolism, and antioxidant production was constructed from the transcriptome GO and KEGG enrichment results with significantly differentially-expressed genes in QTL analysis to determine the effects of metabolic pathways on rice under anaerobic stress. The genes localized with QTL analysis were also analyzed by WGCNA to identify key candidate genes and to perform dominant haplotype analysis.

Energy is the key to seed germination. In the metabolic pathway network, *α-AMY*, *PDC*, *ADH* and other key genes for ATP production all showed increases in expression in response to anaerobic stress, indicating that rice seeds can significantly enhance energy metabolism to promote seed germination after experiencing hypoxia. The expression of these genes increased significantly in HD86 and did not differ significantly in the progeny after anaerobic stress, suggesting that HD86 may elevate the expression of energy metabolism-related genes earlier than NIP in response to anaerobic stress, which is supported by the longer coleoptiles in HD86 compared to NIP. The most relevant plant hormone to the coleoptile is auxin, and the expression of *YUCCA* genes, which are involved in the production of auxin, was not strongly correlated with coleoptile length in this study. However, expression of the auxin downstream gene *EXPA*, which showed a dramatic increase in expression after NIP and HR were subjected to anaerobic stress, showed a strong correlation with coleoptile length. It is possible that *EXPA* genes play a key role in elongation of the coleoptile in NIP compared to HD86 in the late stage. This is similar to a recent study showing that energy metabolism is a pre-condition for the growth of the coleoptile, and that auxin is a determinant of coleoptile length (Nghi et al. 2021). In addition, comparing the expression of genes involved in the production of antioxidant substances (*SOD, POD, CAT*, and *GSH*), the expression of *POD* genes tended to be more obviously up-regulated in both NIP and HR after exposure to anaerobic stress, which could indicate that this enzyme is the main means of alleviating oxidative stress in rice varieties in response anaerobic stress. These results also indicate that antioxidant enzymes positively promote growth of the coleoptile.

The key rice stress tolerance genes *Os05g0498700,* and *Os01g0866100* were identified from the WGCNA and QTL. The Os05g0498700 protein contains the DCD domain, and many similar proteins are associated with plant development and stress (Reis et al. 2016), may affects abscisic acid (ABA) (de Camargos et al. 2019) and gibberellic acid (GA) (Li et al. 1998). Under anaerobic stress conditions, the coleoptile length of the *Os05g0498700* mutant material exceeded that of the wild-type (ZH11), accompanied by significantly elevated expression levels of α-amylase (*Os02g0765600*, *Os08g0473600*) and trehalose-6-phosphatase (*Os09g0369400*) in the mutant compared to the wild type. These findings suggest that the gene may enhance coleoptile length growth by influencing energy utilization. *Os01g0866100* encodes actin, and in a anaerobic environment, the coleoptile of the mutant material also exhibits increased length compared to the wild type. Additionally, the mutant displays significantly higher expression levels of plant hormone-related genes, such as gibberellin (*Os01g0883800*, *Os01g0883800*) and abscisic acid (*Os09g0457100*), when compared to the wild type. These findings suggest that the gene may enhance coleoptile growth by influencing plant hormone pathways. Upon establishing that *Os05g0498700* and *Os01g0866100* potentially regulate coleoptile growth through energy metabolism and plant hormone pathways, we conducted an analysis of the dominant haplotypes of these target genes for their relevance in domestication and breeding utilization. Our investigation revealed that *Os05g0498700* and *Os01g0866100* possess breeding utilization value. To further support our findings, we calculated Pi and Tajima’s D for the target genes, and the results indicated that these genes have been subjected to selective pressures. In this study, we conducted an analysis of the influence of metabolic pathways and key genes on rice coleoptile length. However, it is worth noting that the present evaluation of tolerance to anaerobic germination in rice tends to emphasize coleoptile length excessively. We propose that future research should focus on screening for more robust phenotypes associated with anaerobic stress. Furthermore, it is essential to conduct further investigations on the key genes identified, including exploring their interactions with transcription factors and proteins. This approach will help in gaining a deeper understanding of the molecular mechanisms underlying the hypoxia tolerance of these genes.

## Conclusion

Based on the findings and discussions presented in this paper, it is evident that plant hormones such as ABA and GA play pivotal roles in seed germination and coleoptile length growth. These hormones subsequently up-regulate the expression of genes involved in the energy metabolism pathway, such as amylase, PDC, and ADH, to provide the necessary energy for embryonic growth. Additionally, they contribute to the up-regulation of growth hormone-related genes, such as EXPA, facilitating rapid embryo growth. Specifically, *Os05g0498700* appears to primarily enhance coleoptile length growth by regulating the expression of genes associated with energy synthesis, while *Os01g0866100* promotes coleoptile length growth through its control over plant hormone-related changes.

## Materials and Methods

### Plant Material

‘Haidao86’ (HD86) and ‘Nipponbare’ (NIP) were used as the parents and were crossed to obtain F_1_-generation hybrid plants. Anther culture of F_1_ plants was used to generate a population of 159 doubled haploid (DH) lines for subsequent experiments.

### Determination of Parental Coleoptile Length, Disaccharide Contents, and Related Enzyme Activities

To determine the tolerance to anaerobic germination of the plant material, 100 plump, uniform seeds of the parental lines HD86 and NIP were selected and placed in a 50 °C oven for three days to break dormancy, after which they were surface sterilized in 0.5% sodium hypochlorite solution for 30 min. Three biological replicates of 10 seeds each were set up for anaerobic stress each treatment and the control. Anaerobic stress treatment: A glass tube 12 cm high and 2.5 cm in diameter was filled with distilled water to a depth of 8 cm to simulate anaerobic conditions. Control treatment: seeds were placed in a 9 cm diameter Petri dish lined with two layers of wet filter paper that was kept wet. The experimental conditions were 28℃ and 24 h in darkness, and the lengths of the coleoptiles were measured at different times.

The contents of the sugar compounds, including d-glucose, fructose, d-galactose, trehalose, maltose and sucrose, were measured in the control and anaerobic environments when the differences in the lengths of the parental line coleoptiles were the greatest (day 4). The coleoptile α-amylase (α-AL) and pyruvate decarboxylase (PDC) activities were measured simultaneously. All of the above experiments were in three biological replicates. The enzyme activity kits were purchased from Suzhou Kemin and used as directed by the manufacturer. Enzyme activities were calculated with the following formulas:

$${\text{PDC }}\left( {{\text{nmol}}/{\text{min}}/{\text{g fresh weight}}} \right)\, = \,{16}0{8}\, \times \,\Delta {\text{A}}\, \div \,{\text{W}}.$$

$$\alpha - {\text{AL }}\left( {{\text{mg}}/{\text{min}}/{\text{g fresh weight}}} \right)\, = \,{1}.{612}\, \times \,\left( {\Delta {\text{A}}\, + \,0.{1778}} \right)\, \div \,{\text{W}}.$$

W: sample mass in grams; ΔA: difference in absorbance value.

### Transcriptome Sequencing and Analysis in the Parental Cultivars and the DH Progeny

Total RNA was extracted from day-4 coleoptiles of NIP (parental cultivar) and HR (DH progeny), which are strongly tolerant of anaerobic conditions, and HD86 (parental cultivar) and NHR (DH progeny), which are weakly tolerant to hypoxia, for transcriptomic analyses. Under anaerobic stress, HR is the material with the longest coleoptile length, while NHR is the shortest. The raw data was filtered and subjected to quality control using fastp software to obtain clean data. The clean reads were aligned to the reference genome using hisat2 software, and the clean reads were assembled using StringTie. Gene and transcript expression read counts and FPKM (fragments per kilobase of transcript per million mapped reads) were calculated using featureCounts and StringTie software. The differentially-expressed genes (DEGs) were identified using |log2 Fold-change|≥ 1 and q < 0.05 as the screening criteria. GO and KEGG enrichment analysis and WGCNA analysis of the DEGs was performed using R (version 4.1.1). WGCNA analysis enabled the assignment of genes to different modules based on the gene expression, and the different modules were associated with coleoptile length. The key genes in the modules were identified by the degree of association between genes.

### Statistics on Coleoptile Length and Seedling Survival Rate in the DH Population, and QTL Localization

Determination of the length of the coleoptile on the population material with reference to ‘Determination of parental coleoptile length, disaccharide contents, and related enzyme activities. The rice 40 K SNP array was used for genotype identification. First, high quality SNP markers (missing data < 5%) that showed polymorphism between the parental lines were selected from the 40 K SNPs for genetic map construction and QTL localization analysis. The BIN function of ICIMapping (version 4.2) (Cao et al. 2019) was used to remove redundant markers. The remaining SNP markers were used for genetic linkage map construction, and the BIP function was applied to QTL localization for traits related to tolerance to anaerobic germination at germination with a QTL screening threshold of LOD ≥ 2.5.

$${\text{Anaerobic response index}}\, = \,{\text{length of embryo in the treated group}}{-\!\!-}{\text{length of embryo in the control group}}$$

$${\text{coleoptile length ratio}}\, = \,{\text{coleoptile length in the treatment group}}/{\text{coleoptile length in the control group}}.$$

### Transcriptome and QTL Association Analyses

The key pathways of tolerance to anaerobic germination in rice were identified by transcriptome enrichment analysis and differential gene expression changes in the QTL mapping intervals, and the pathway changes in tolerance to anaerobic germination in rice were resolved. Key genes for tolerance to anaerobic germination were identified by WGCNA analysis and QTL localization. The identified key genes were subjected to haplotype analysis. A total of 498 rice accessions (Islam et al. 2022b) were selected from the 3000 Rice Genomes Project for the determination of coleoptile length (Wang et al. 2018). Rice haplotypes with a number of phenotypes greater than 10 were selected to determine whether the selected key genes were associated with rice coleoptile length.

### Quantitative Real-Time PCR Analysis

To determine whether the RNA sequencing data accurately represented relative gene expression, six DEGs were selected at random for qRT-PCR analysis. The PCR reactions were performed in an ABI 7500 Real-Time PCR system using a qRT-PCR kit (Vazyme Biotech). *OsACT11* was used as an internal control for normalization of target gene expression. The primers used in the qRT-PCR experiment are listed in Table S7.

### Genetic Transformation of Candidate Genes

To construct a gene knockout vector, a target site specific to the cDNA sequence of the candidate gene was designed. Agrobacterium-mediated genetic transformation was employed to introduce this construct into Zhonghua11 (ZH11). Subsequently, the coleoptile length of transgenic positive plants subjected to hypoxia stress was assessed, with each gene being evaluated with 20 biological replicates.

### Phenotype is Associated with Haplotype

The identified key genes underwent haplotype analysis. A dataset comprising 498 rice accessions, as reported by Islam et al. (2022), was selected from the 3,000 Rice Genomes Project (Wang et al. 2018) for coleoptile length determination. Rice haplotypes exhibiting a phenotype count exceeding 30 were chosen to investigate whether the selected key genes exhibited associations with rice coleoptile length. The analysis of rice germplasm resource haplotypes was conducted using RFGB (https://www.rmbreeding.cn/), and dominant haplotypes were ascertained in conjunction with coleoptile lengths.

### Haplotype Derivation Analysis and Breeding Utilization Analysis of Candidate Gene

The candidate genes were created using the coding sequence (CDS) and single nucleotide polymorphisms (SNPs) found within the 2-kilobase (kb) promoter sequence, respectively. Variety groups of these candidate genes were established based on SNP genotypes sourced from published wild rice germplasm resources and 3 k resources available in the MBKbase database (http://www.mbkbase.org/rice/customGT), employing the following parameters: ‘ALT ≥ 5%, Missing ≤ 20%’. The haplotype network for the candidate genes was generated using the minimum spanning tree method in Popart (version 1.7).

### Selection Analysis of Key Genes

Single nucleotide polymorphisms (SNPs) within the gene region and within a 20-kilobase (kb) region both upstream and downstream of the gene were extracted from datasets consisting of 3 k cultivated rice genotypes and 118 wild rice genotypes (MBKbase database), respectively. To analyze the data, we utilized Filter of TASSEL 5 software (version 5.2.82), which enabled us to calculate population genetic parameters, including nucleotide polymorphisms (π/pi) and Tajima’s D.

### Supplementary Information

Additional file 1.

Additional file 2.

## Data Availability

RNA-seq data were deposited to the SRA database of NCBI (PRJNA1043854).

## Abbreviations

WGCNA: Weighted gene co-expression network analysis
QTL: Quantitative trait locus
DH: Doubled-haploid
DEGs: Differentially-expressed genes
GO: Gene ontology
KEGG: Kyoto Encyclopedia of Genes and Genomes
PDC: Pyruvate decarboxylase
ADH: Alcohol dehydrogenase
POD: Peroxidase
ROS: Reactive oxygen species
ATP: Adenosine triphosphate
SOD: Superoxide dismutase
CAT: Catalase

## References

[CR1] Cao Z, Guo Y, Yang Q (2019). Genome-wide identification of quantitative trait loci for important plant and flower traits in petunia using a high-density linkage map and an interspecific recombinant inbred population derived from *Petunia integrifolia* and *P axillaris*. Hortic Res.

[CR2] Chaudhary C, Sharma N, Khurana P (2021). Decoding the wheat awn transcriptome and overexpressing TaRca1β in rice for heat stress tolerance. Plant Mol Biol.

[CR3] de Camargos LF, Fraga OT, Oliveira CC (2019). Development and cell death domain-containing asparagine-rich protein (DCD/NRP): an essential protein in plant development and stress responses. Theor Exp Plant Physiol.

[CR4] Derakhshani B, Jafary H, Maleki Zanjani B (2020). Combined QTL mapping and RNA-Seq profiling reveals candidate genes associated with cadmium tolerance in barley. PLoS ONE.

[CR5] Hsu S-K, Tung C-W (2015). Genetic mapping of anaerobic germination-associated QTLs controlling coleoptile elongation in rice. Rice.

[CR6] Islam MR, Naveed SA, Zhang Y (2022). Identification of candidate genes for salinity and anaerobic tolerance at the germination stage in rice by genome-wide association analyses. Front Genet.

[CR7] Langfelder P, Horvath S (2008). WGCNA: an R package for weighted correlation network analysis. BMC Bioinformatics.

[CR8] Lee K-W, Chen PW, Yu S-M (2014). Metabolic adaptation to sugar/O_2_ deficiency for anaerobic germination and seedling growth in rice. Plant Cell Environ.

[CR9] Li HY, Guo ZF, Zhu YX (1998). Molecular cloning and analysis of a pea cDNA that is expressed in darkness and very rapidly induced by gibberellic acid. Mol Gen Genet.

[CR10] Lv Y, Shao G, Jiao G (2021). Targeted mutagenesis of POLYAMINE OXIDASE 5 that negatively regulates mesocotyl elongation enables the generation of direct-seeding rice with improved grain yield. Mol Plant.

[CR11] Mondal S, Khan MIR, Entila F (2020). Responses of AG1 and AG2 QTL introgression lines and seed pre-treatment on growth and physiological processes during anaerobic germination of rice under flooding. Sci Rep.

[CR12] Muhammad II, Kong SL, Akmar Abdullah SN, Munusamy U (2019). RNA-seq and ChIP-seq as complementary approaches for comprehension of plant transcriptional regulatory mechanism. Int J Mol Sci.

[CR13] Nghi KN, Tagliani A, Mariotti L (2021). Auxin is required for the long coleoptile trait in japonica rice under submergence. New Phytol.

[CR14] Pradhan SK, Pandit E, Nayak DK (2019). Genes, pathways and transcription factors involved in seedling stage chilling stress tolerance in indica rice through RNA-Seq analysis. BMC Plant Biol.

[CR15] Pucciariello C (2020). Molecular mechanisms supporting rice germination and coleoptile elongation under low oxygen. Plants.

[CR16] Reis PAB, Carpinetti PA, Freitas PPJ (2016). Functional and regulatory conservation of the soybean ER stress-induced DCD/NRP-mediated cell death signaling in plants. BMC Plant Biol.

[CR17] Septiningsih EM, Ignacio JCI, Sendon PMD (2013). QTL mapping and confirmation for tolerance of anaerobic conditions during germination derived from the rice landrace Ma-Zhan Red. Theor Appl Genet.

[CR18] Sevanthi AM, Sinha SKVS (2021). Integration of dual stress transcriptomes and major QTLs from a pair of genotypes contrasting for drought and chronic nitrogen starvation identifies key stress responsive genes in rice. Rice.

[CR19] Siddiqui MN, Mostofa MG, Rahman MM (2021). Glutathione improves rice tolerance to submergence: insights into its physiological and biochemical mechanisms. J Biotechnol.

[CR20] Takahashi H, Greenway H, Matsumura H (2014). Rice alcohol dehydrogenase 1 promotes survival and has a major impact on carbohydrate metabolism in the embryo and endosperm when seeds are germinated in partially oxygenated water. Ann Bot.

[CR21] Wang W, Mauleon R, Hu Z (2018). Genomic variation in 3,010 diverse accessions of Asian cultivated rice. Nature.

[CR22] Wen J, Jiang F, Weng Y (2019). Identification of heat-tolerance QTLs and high-temperature stress-responsive genes through conventional QTL mapping, QTL-seq and RNA-seq in tomato. BMC Plant Biol.

[CR23] Würschum T (2012). Mapping QTL for agronomic traits in breeding populations. Theor Appl Genet.

[CR24] Xin F, Xiao X, Dong J (2020). Large increases of paddy rice area, gross primary production, and grain production in northeast China during 2000–2017. Sci Total Environ.

[CR25] Yu S-M, Lee H-T, Lo S-F, Ho T-HD (2021). How does rice cope with too little oxygen during its early life?. New Phytol.

[CR26] Zhang H, Zhang J, Xu Q (2020). Identification of candidate tolerance genes to low-temperature during maize germination by GWAS and RNA-seqapproaches. BMC Plant Biol.

[CR27] Zhu G, Li W, Zhang F, Guo W (2018). RNA-seq analysis reveals alternative splicing under salt stress in cotton. Gossypium Davidsonii BMC Genomics.

