# Supplementary material for: Identification of Key Genes and Pathways for Anaerobic Germination Tolerance in Rice Using Weighted Gene Co-Expression Network Analysis (WGCNA) in Association with Quantitative Trait Locus (QTL) Mapping
Source: Rice (N Y). 2024 May 31;17:37. doi: 10.1186/s12284-024-00714-y (PMC11143092; doi:10.1186/s12284-024-00714-y)
Supplement: Supplementary file 1 — Additional file 1. [file 12284_2024_714_MOESM1_ESM.ppt]

## Slide 1
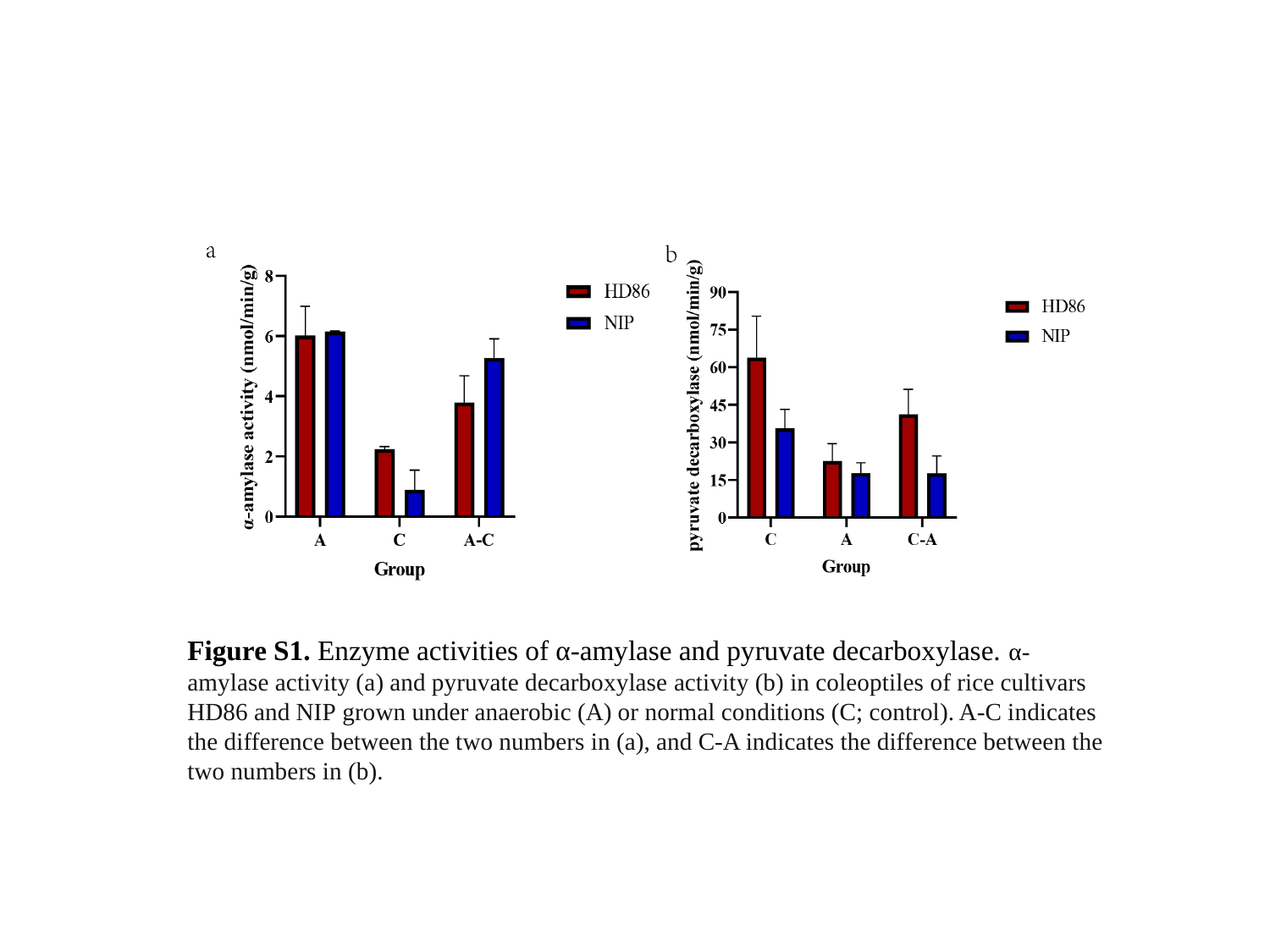

Figure S1. Enzyme activities of α-amylase and pyruvate decarboxylase. α-amylase activity (a) and pyruvate decarboxylase activity (b) in coleoptiles of rice cultivars HD86 and NIP grown under anaerobic (A) or normal conditions (C; control). A-C indicates the difference between the two numbers in (a), and C-A indicates the difference between the two numbers in (b).

## Slide 2
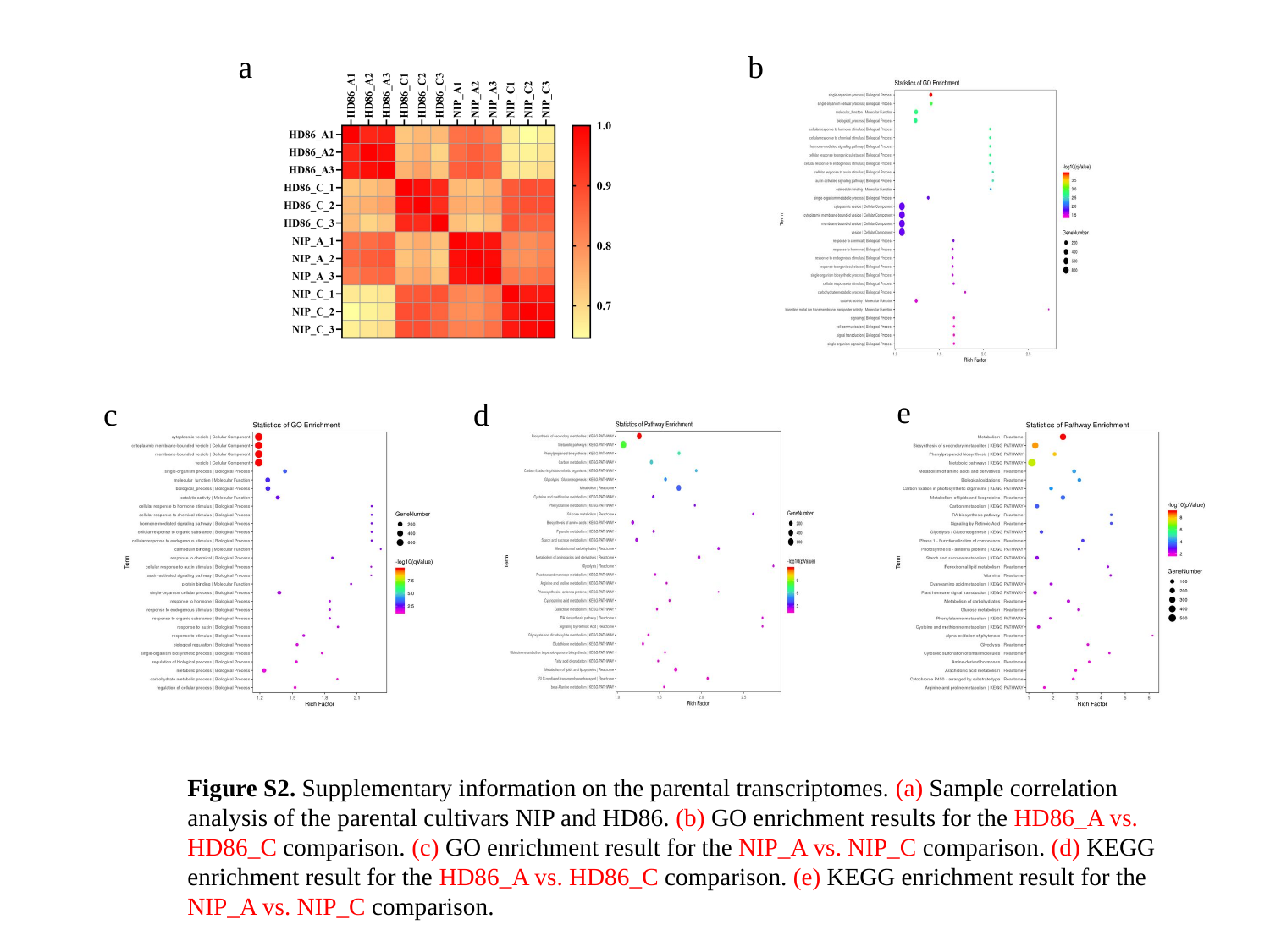

a
b
e
c
d
Figure S2. Supplementary information on the parental transcriptomes. (a) Sample correlation analysis of the parental cultivars NIP and HD86. (b) GO enrichment results for the HD86_A vs. HD86_C comparison. (c) GO enrichment result for the NIP_A vs. NIP_C comparison. (d) KEGG enrichment result for the HD86_A vs. HD86_C comparison. (e) KEGG enrichment result for the NIP_A vs. NIP_C comparison.

## Slide 3
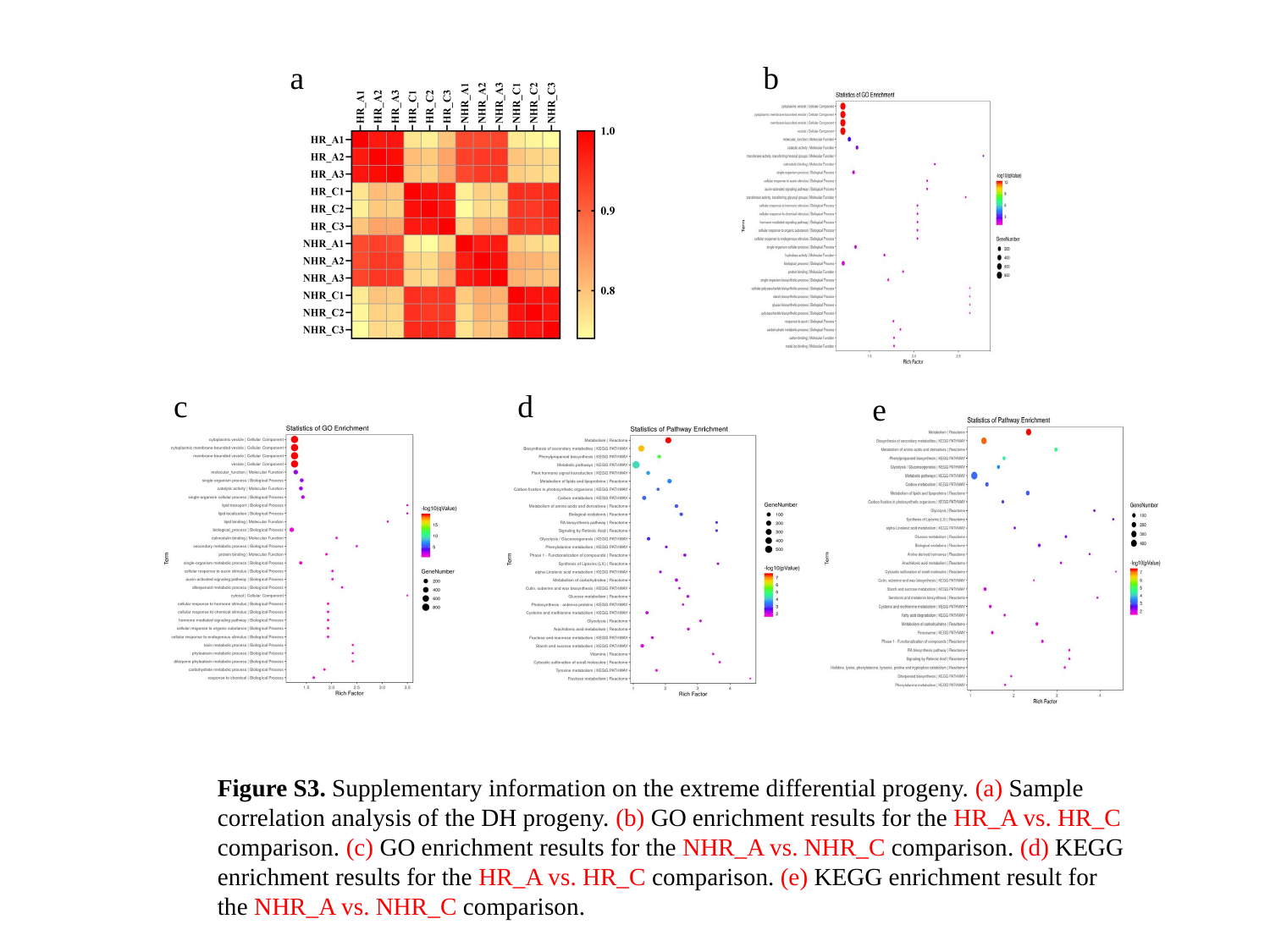

a
b
c
d
e
Figure S3. Supplementary information on the extreme differential progeny. (a) Sample correlation analysis of the DH progeny. (b) GO enrichment results for the HR_A vs. HR_C comparison. (c) GO enrichment results for the NHR_A vs. NHR_C comparison. (d) KEGG enrichment results for the HR_A vs. HR_C comparison. (e) KEGG enrichment result for the NHR_A vs. NHR_C comparison.

## Slide 4
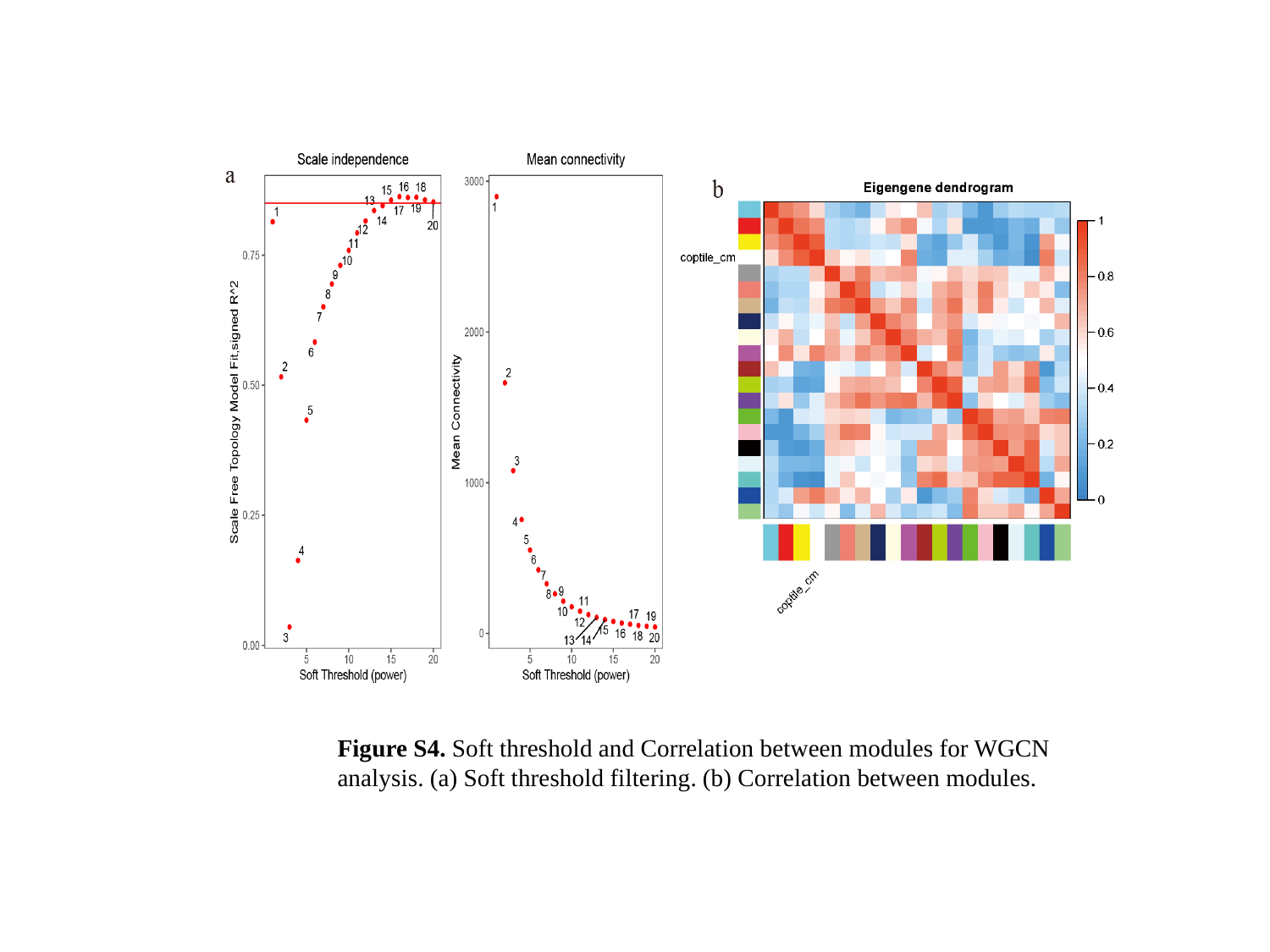

Figure S4. Soft threshold and Correlation between modules for WGCN analysis. (a) Soft threshold filtering. (b) Correlation between modules.

## Slide 5
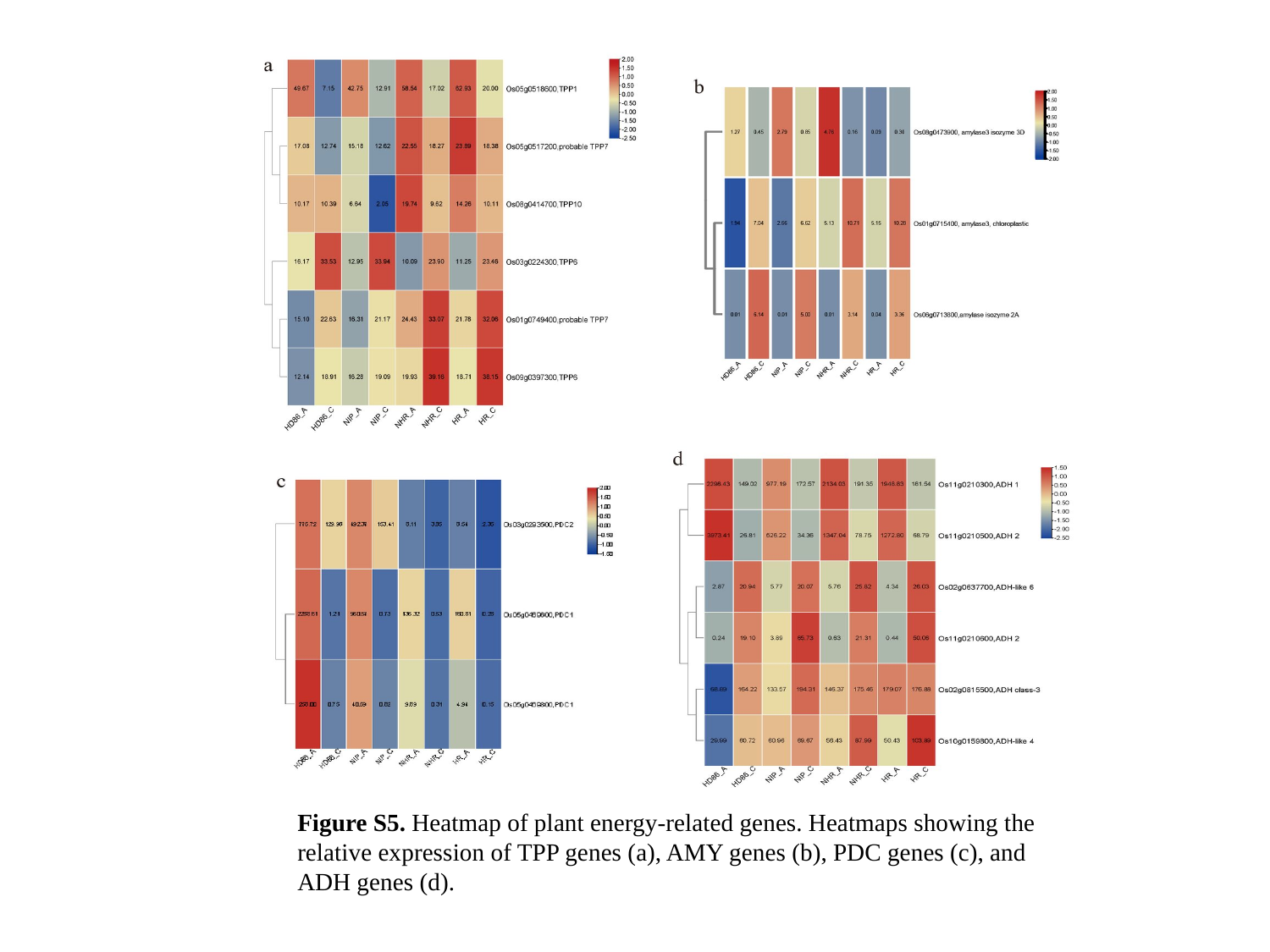

Figure S5. Heatmap of plant energy-related genes. Heatmaps showing the relative expression of TPP genes (a), AMY genes (b), PDC genes (c), and ADH genes (d).

## Slide 6
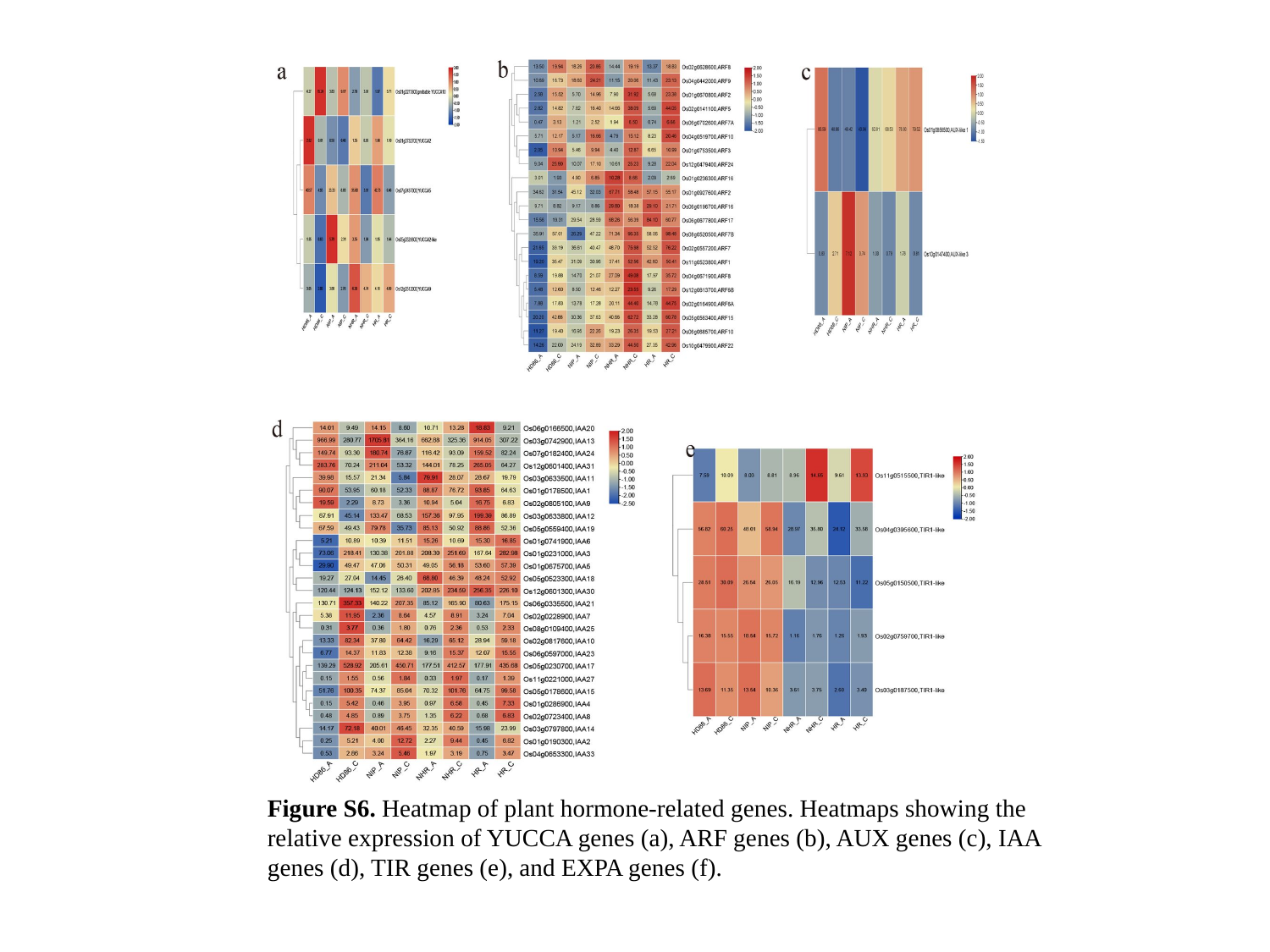

Figure S6. Heatmap of plant hormone-related genes. Heatmaps showing the relative expression of YUCCA genes (a), ARF genes (b), AUX genes (c), IAA genes (d), TIR genes (e), and EXPA genes (f).

## Slide 7
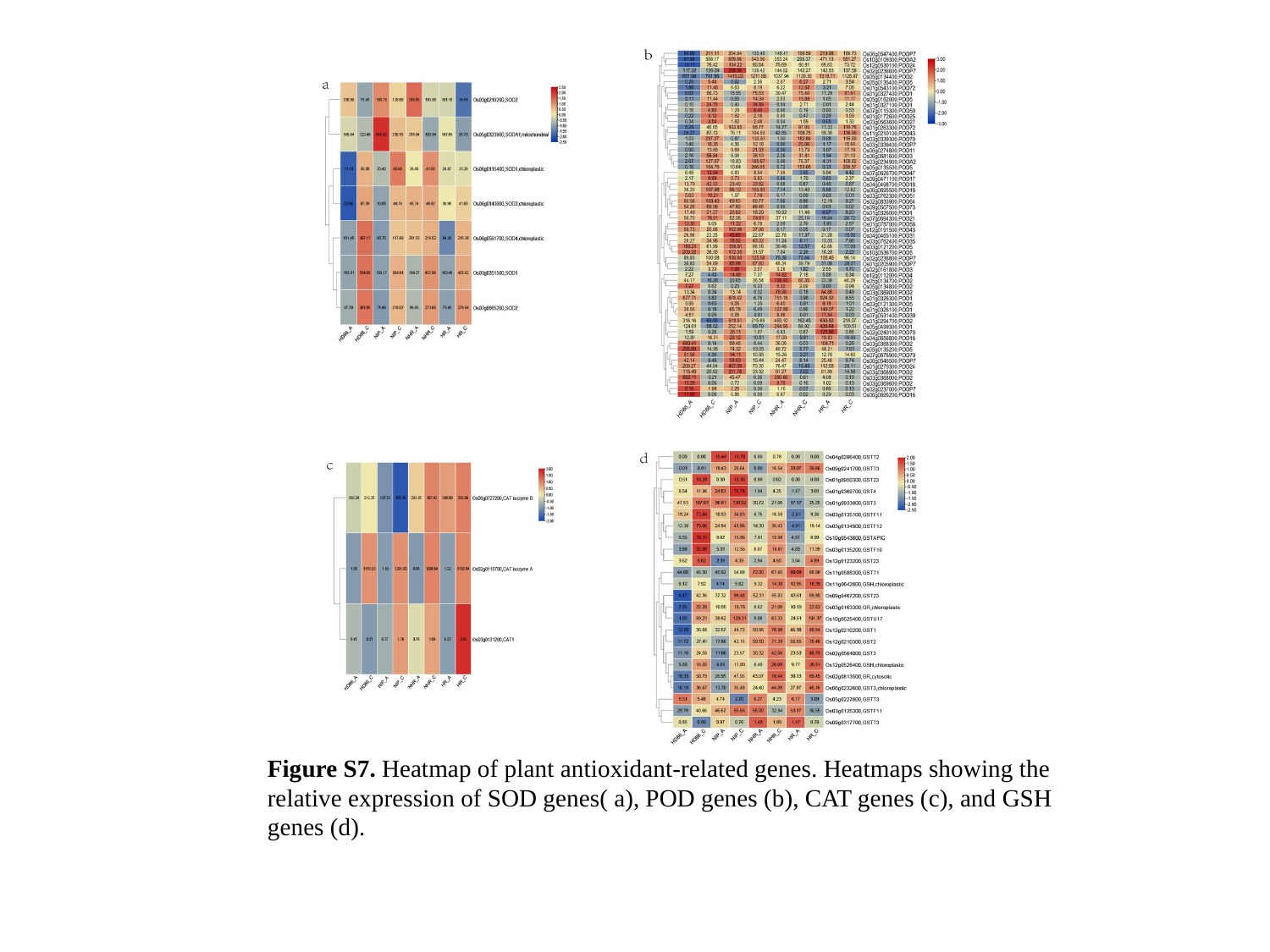

Figure S7. Heatmap of plant antioxidant-related genes. Heatmaps showing the relative expression of SOD genes( a), POD genes (b), CAT genes (c), and GSH genes (d).
